# Proportion and antibiogram of methicillin-resistant *Staphylococcus aureus* (MRSA) in Africa: a systematic review and meta-analysis

**DOI:** 10.1186/s13756-025-01687-3

**Published:** 2026-01-21

**Authors:** Ahmed Azzam, Heba Khaled, Ahmed Salem, Muhamad Sayed, Abdelmarouf Mohieldein, Mohamed S. Elsayed, Enas Mohamed Lotfy, Hend H. A. M. Abdullah, Fatma E. Hassan, Hassan Marei, Nouran Hassan, Elham Abdulnaby, Gellan Alaa Mohamed Kamel, Ismael Osman, Mohamed Ahmed Reda, Dina Ismail, Mahmoud Nazih, Haitham Salem, Amar Basil, Dina Rady

**Affiliations:** 1 Department of Microbiology and Immunology, Faculty of Pharmacy, Capital University (formerly Helwan University), Cairo, Egypt; 2 Department of Biochemistry, Faculty of Pharmacy, Cairo University, Cairo, Egypt; 3 Tanta Health Insurance Hospital, Gharbia, Egypt; 4 Minya Chest Hospital, Minya, Egypt; 5 Department of Medical Laboratories, College of Applied Medical Sciences, Qassim University, Buraidah, Saudi Arabia; 6 Pharmacology Department, Faculty of Pharmacy, Merit University, Sohag, Egypt; 7 Department of Public Health Nursing, College of Nursing, Northern Border University, Arar, Saudi Arabia; 8 Department of Parasitology and Animal Diseases, Veterinary Research Institute, National Research Centre, Giza, Egypt; 9 Department of Physiology, General Medicine Practice Program, Batterjee Medical College, Jeddah, 21442 Saudi Arabia; 10 Medical Physiology Department, Faculty of Medicine, Kasr Al-Ainy, Cairo University, Giza, 11562 Egypt; 11 Cairo University Student Hospital, Giza, Egypt; 12 Department of Anatomy, Histology and Pathology, Faculty of Pharmacy, October University for Modern Sciences & Arts, Cairo, Egypt; 13 Department of Central Labs, National Nutrition Institute, Cairo, Egypt; 14 Department of Pharmacology and Toxicology, College of Pharmacy, Uruk University, Baghdad, Iraq; 15 Internal Medicine Resident, Shubra Health Insurance Hospital, Cairo, Egypt; 16 Department of Cardiothoracic Surgery, Ain Shams University, Cairo, Egypt; 17 Al Karkh General Hospital, Baghdad, Iraq; 18 Al Ryada University for Science and Technology, Sadat City, Menoufia 16504 Egypt; 19 Department of Clinical Pharmacy, Faculty of Pharmacy, Ahram Canadian University, 6th of October City, Giza, 12566 Egypt; 20 Faculty of Medicine, Ain Shams University, Cairo, Egypt; 21 Bashir Hospital, Amman, Jordan; 22 Department of Oral Biology, Faculty of Dentistry, Cairo University, Cairo, Egypt

**Keywords:** Epidemiology, Surveillance, Antimicrobial resistance, *Staphylococcus aureus*, MecA, Nosocomial infections, Africa

## Abstract

**Background:**

Methicillin-resistant *Staphylococcus aureus* (MRSA) is a major public health concern, particularly in resource-limited settings such as Africa. This meta-analysis aimed to determine the proportion of MRSA among *S. aureus* isolates from patients with confirmed infections and to assess associated antibiotic resistance profiles across the continent.

**Methods:**

A comprehensive literature search was conducted in African Journals Online, African Index Medicus, PubMed, Scopus, Google Scholar, and Web of Science for studies published between January 1, 2013, and June 5, 2024. Primary studies were included if they reported MRSA proportion or resistance profiles in Africa, employed reliable detection techniques, and analyzed clinical specimens from infected patients. Statistical analyses were performed using the meta package in R software, applying a random-effects model. A *p*-value of < 0.05 was considered statistically significant.

**Results:**

This meta-analysis included 191 studies, encompassing 40,979 *S. aureus* isolates. Nigeria contributed the highest number of studies (*n* = 29), followed by Egypt (*n* = 26). The vast majority of studies (*n* = 186) were based on hospital settings. The pooled proportion of MRSA in Africa was 42.2% (95% CI 38.7–45.6). By detection method, proportion was 41.4% for *mecA*, 42.8% for the cefoxitin disc method, and 39.1% for the oxacillin disc method, with no significant differences observed (*p* = 0.8). Regionally, Northern Africa had a significantly higher proportion of 56.2% (95% CI 49.3–62.9) compared with 36.7% (95% CI 33.2–40.4) in Sub-Saharan Africa (*p* < 0.001). At the country level, Eritrea reported the highest proportion (71.8%), followed by Egypt (61.8%), while the lowest rates were observed in Malawi (7.0%) and Gabon (8.2%). Regarding MRSA resistance profiles, linezolid (3.4%) and vancomycin (4.7%) showed the lowest resistance rates, whereas higher rates were noted for fusidic acid (11.6%), rifampin (28.4%), clindamycin (40.4%), trimethoprim–sulfamethoxazole (54.5%), and tetracycline (60.2%). Limited data were available for telavancin, dalbavancin, oritavancin, tedizolid, ceftaroline, mupirocin, and daptomycin.

**Conclusion:**

The proportion of MRSA in Africa remains high at 42.2%, with marked regional disparities. Although resistance rates for linezolid and vancomycin are relatively low, they surpass global averages, raising concerns about emerging resistance. Alarmingly high resistance rates to several other antibiotics further underscore the urgent need for targeted interventions and continuous surveillance.

**Supplementary Information:**

The online version contains supplementary material available at 10.1186/s13756-025-01687-3.

## Introduction

The World Health Organization (WHO) recognizes antimicrobial resistance (AMR) as one of the top ten global public health threats, highlighting the critical need for action [1]. Among the most concerning contributors to this crisis is methicillin-resistant *Staphylococcus aureus* (MRSA), which continues to pose a significant nosocomial threat with far-reaching public health implications [2, 3]. Several studies have demonstrated that MRSA is associated with higher morbidity and mortality rates, increased hospital costs, and longer hospital stays compared to methicillin-susceptible *Staphylococcus aureus* (MSSA) [4–7]. These findings highlight the critical need to address MRSA, as evidenced by its designation as a “serious threat” in the Centers for Disease Control and Prevention’s (CDC) 2019 Antibiotic Resistance Threat Report and its inclusion on the WHO 2024 high-priority pathogen list for urgent antibiotic development [2, 3]. The urgency of addressing MRSA lies in its intrinsic resistance to beta-lactam antibiotics. This resistance significantly restricts treatment options, delays the timely initiation of effective antimicrobial therapy, and frequently leads to poorer clinical outcomes. It is predominantly driven by the *mecA* gene, which encodes PBP2a—a unique penicillin-binding protein with substantially reduced affinity for beta-lactam antibiotics [8].

Quantifying the burden of MRSA is essential for assessing its global impact on public health and healthcare systems. Accurate data are crucial for guiding resource allocation, developing effective infection control strategies, and identifying regions that require urgent attention. Globally, MRSA proportion is influenced by multiple factors, including antibiotic misuse, inadequate infection prevention measures, and socioeconomic inequalities, all of which contribute to marked regional variation [9–14].

These challenges are particularly severe in resource-limited settings such as Africa, where the burden of MRSA and AMR is compounded by limited comprehensive data, widespread healthcare inequalities, inadequate antibiotic stewardship programs (ASPs), insufficient infection control measures, pervasive antibiotic self-medication, and constrained public health funding [15–22].

To the best of our knowledge, there are no current pooled data on MRSA proportion in Africa. To address this gap, We conducted a meta-analysis to quantify the proportion of MRSA among clinically isolated *S. aureus* and to examine its antimicrobial resistance profile against key treatment options. Our findings provide a comprehensive overview of MRSA epidemiology across the continent, filling critical knowledge gaps and offering actionable insights to inform policy development, strengthen antimicrobial stewardship, and enhance infection control measures.

## Methods

### Search strategy

A comprehensive literature search was conducted using the following databases: African Journals Online, African Index Medicus, PubMed, Scopus, Google Scholar, and Web of Science. The search covered the period from January 1, 2013, to June 5, 2024, to include up-to-date data and to reflect current trends in MRSA proportion. The detailed search strategy is presented in Table S1. It was adapted to align with the specific requirements and functionalities of each database. An example of the detailed search strategy for the Scopus and PubMed databases is provided in Table S2. This study followed the PRISMA (Preferred Reporting Items for Systematic Reviews and Meta-Analyses) guidelines [23]. Table S3 presents the PRISMA Main Checklist (27-item checklist).

### Eligibility criteria

#### Inclusion criteria

Studies were included if they met the following criteria: (1) they were primary studies reporting the proportion of MRSA or its antimicrobial resistance profile in any African country; (2) adherence to CLSI or EUCAST breakpoints for interpreting antimicrobial susceptibility results; (3) use of valid detection methods for MRSA; (4) clinical specimens collected from patients; and (5) publication between January 1, 2013, and June 5, 2024.

In cases where studies employed multiple detection methods for MRSA, preference was given to the most sensitive and specific techniques. PCR detection of *mecA*, widely regarded as the gold standard, was prioritized, followed by the rapid latex agglutination (RLAT) PBP2a assay, cefoxitin disc diffusion test (CDD), and, finally, the oxacillin disc diffusion test (ODD) [24–26].

Additionally, in studies that included both human and non-human data (e.g., animals or livestock), only the data derived from human clinical samples were included, in line with our predefined inclusion criteria.

#### Exclusion criteria

Studies were excluded if they met any of the following criteria: (1) they were not conducted in Africa, (2) involved specimens from food, animals, or healthy individuals for screening purposes (e.g., nasal or rectal swabs for detecting carriage in asymptomatic individuals), (3) involved pre-selection of multidrug-resistant *S. aureus* isolates, which could bias MRSA proportion estimates; or (4) were literature reviews, preprints, or conference abstracts.

Five authors (H.K., M.N., A.S., M.S., and A.M.) selected the included articles based on the previously mentioned eligibility criteria, which were then cross-checked by another group of five authors (M.E., E.L., H.A., F.H., and H.M.).

### Data extraction

Data extraction was independently performed by five investigators (N.H., E.A., G.H., I.O., and M.R.) and subsequently cross-verified by five additional reviewers (D.I., D.R., A.A., F.H., and H.A.) to ensure accuracy. For each included study, the following information was collected: the first author’s last name, study period, country, African region, type of infection, patients’ age categories, total *Staphylococcus aureus* sample size, number of reported MRSA cases, infection source (community-acquired, nosocomial, inpatient, or outpatient), sample collection setting (hospital setting, private lab, or other sources), specimen type, and method used for MRSA detection, and the antibiogram of MRSA isolates for the antibiotics linezolid, vancomycin, ceftaroline, telavancin, clindamycin, trimethoprim-sulfamethoxazole (TMP-SMX), rifampin, tetracycline, dalbavancin, oritavancin, daptomycin, tedizolid, fusidic acid, and mupirocin. These antibiotics are widely used in the management of MRSA infections [27].

### Quality assessment

The quality of the included studies was meticulously assessed by three reviewers (H.S., M.N., and A.S.) using the Joanna Briggs Critical Appraisal Checklist for Prevalence Studies [27]. This assessment was subsequently verified by three additional reviewers (A.B., E.L., and H.A.). The original checklist items are detailed in Table S4, with a cutoff score of 5 out of 8 set to indicate that a study meets the threshold for fair quality. Question 9 was excluded from the assessment as it was deemed not relevant to this study. This checklist systematically evaluated key aspects, including the appropriateness of the sampling frame, sampling methods, and sample size; the description of study settings; the adequacy of data analysis coverage; the use of validated methods for MRSA identification; the standardization of MRSA detection methods; and the suitability of the statistical analysis.

### Data synthesis

A meta-analysis of MRSA proportion was conducted using the meta package in R, employing a random-effects model based on the inverse variance method. Pre-specified subgroup analyses were conducted based on detection methods, infection types, African regions, and individual African countries. Furthermore, the resistance profiles of MRSA isolates were assessed for the following antibiotics: linezolid, vancomycin, ceftaroline, telavancin, clindamycin, trimethoprim, rifampin, tetracycline, dalbavancin, oritavancin, daptomycin, tedizolid, fusidic acid, and mupirocin. The results were presented as pooled proportion percentages, along with 95% confidence intervals (CIs). Heterogeneity was assessed using I-squared (I^2^) to evaluate the variation between studies. I^2^ values above 75% were considered indicative of high heterogeneity. Sensitivity analyses were performed using a leave-one-out method to assess the robustness of the findings.

## Results

### Characteristics of included studies

**Fig. 1:**
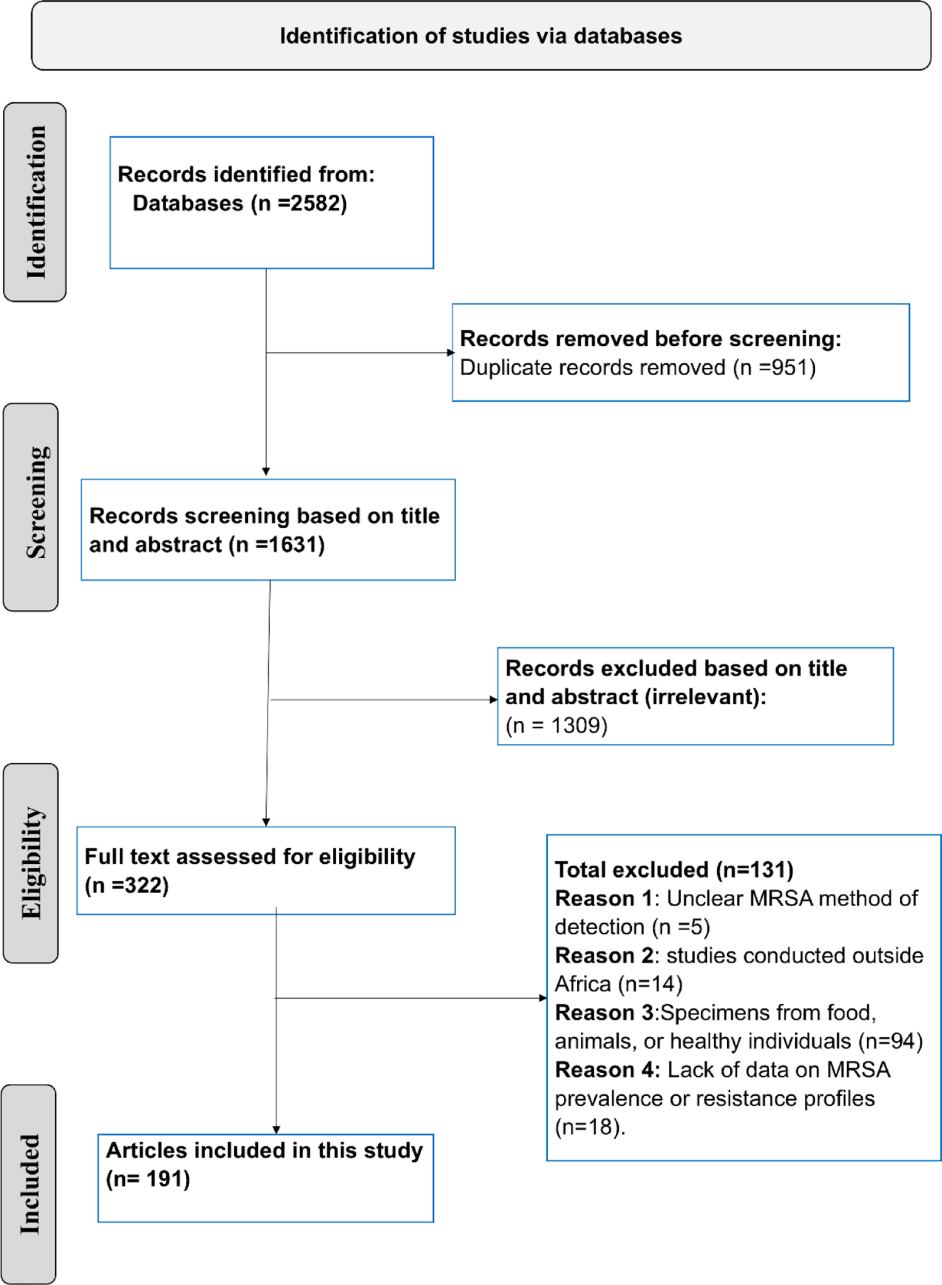
PRISMA flow diagram illustrating the selection process for the included articles

**Fig. 2:**
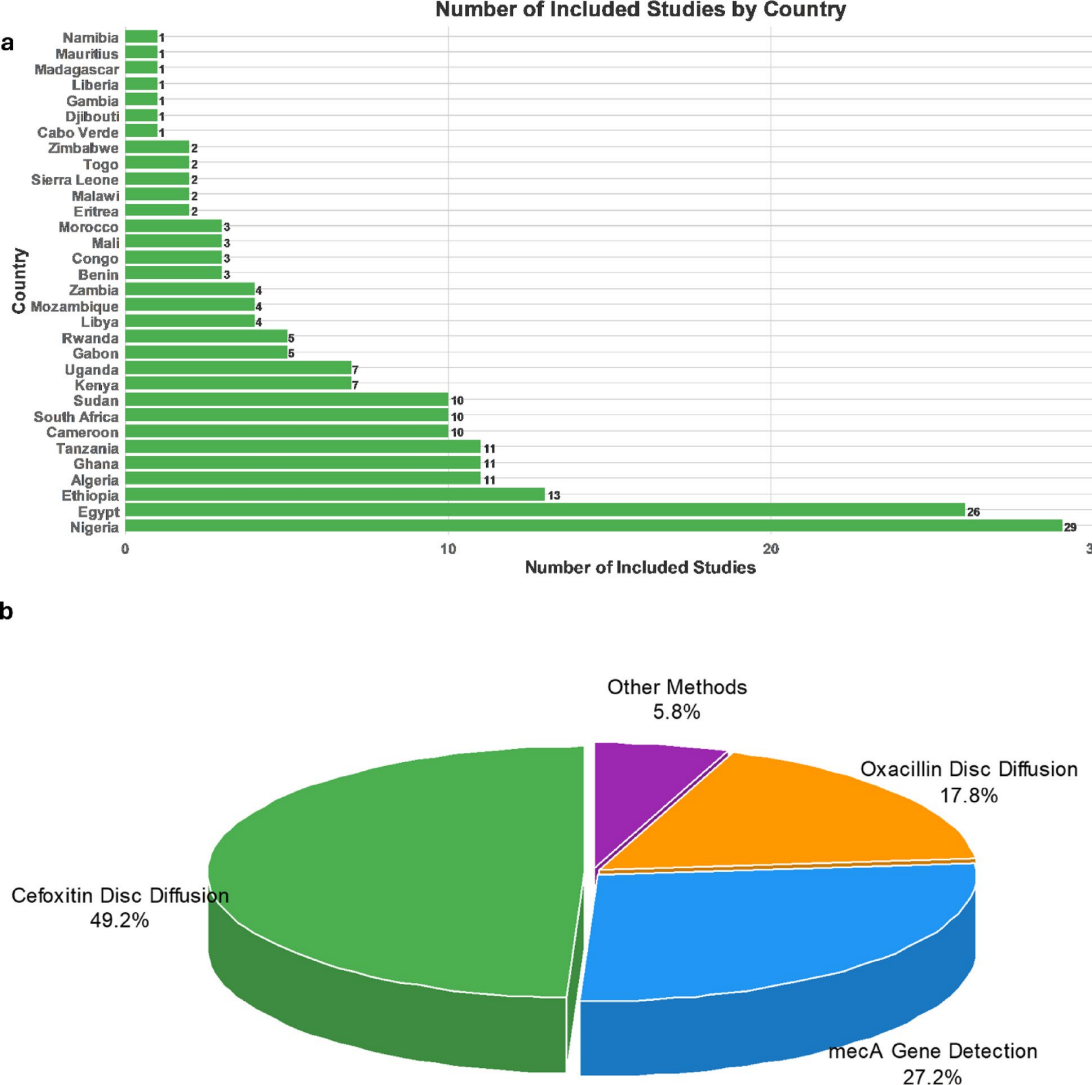
Overview of the distribution of included studies by country and diagnostic methods for MRSA detection. **a** The number of studies included from each country, with Nigeria contributing the highest number (*n* = 29), followed by Egypt (*n* = 26). **b** The percentage distribution of MRSA diagnostic methods used in the included studies. The vast majority of studies relied on cefoxitin disc diffusion for reporting MRSA detection (49.2%). Other methods include oxacillin broth microdilution, oxacillin testing with the E-test, PBP2a assay, cefoxitin testing with the E-test, and the Oxacillin Resistance Screening Agar Base method

This meta-analysis included 191 studies out of 2,582 screened articles conducted across various African countries, as shown in Fig. 1. Of these, 55 studies did not report patient age data, while 136 studies provided age-related information. Among the 136 studies, 84 included patients across all age categories, while the remaining studies focused on neonates, pediatric populations, or adults. Regarding diagnostic methods for MRSA, 94 studies utilized the CDD method, 52 relied on *mecA* gene detection, and 34 employed the ODD test. Additionally, one study used MICs of oxacillin determined by broth microdilution [28], two studies utilized oxacillin testing with the E-test [29, 30], three studies employed the RLAT method [31–33], one utilized cefoxitin testing with the E-test [30], and four applied the Oxacillin Resistance Screening Agar Base method for diagnosis [34–37]. The vast majority of studies (*n* = 186) involved sample collection from hospital settings, while the remaining five included samples collected from private laboratories or other sources [38–42]. Regarding geographic distribution, most studies (*n* = 137) were conducted in Sub-Saharan African countries, with Nigeria contributing the largest share (*n* = 29). In Northern Africa, a total of 54 studies were carried out, with Egypt accounting for the majority (*n* = 26). All studies were conducted within a single country, except for one that encompassed six Sub-Saharan African nations [42]. The number of included studies by country is illustrated in Fig. 2. Regarding the quality of studies, all the included articles received a score of 5 or above, which was deemed to indicate fair quality. The characteristics of the included studies, along with the quality assessment, are presented in Table S5.

### The overall proportion of MRSA in Africa

A total of 191 studies, encompassing 40,979 isolates, were included in this meta-analysis, with an overall pooled MRSA proportion of 42.2% (95% CI 38.7–45.6). Among these, 94 studies employed the CDD method for MRSA detection, reporting a pooled proportion of 42.8% (95% CI 37.2–48.4). Additionally, 52 studies diagnosed MRSA by detecting the *mecA* gene, yielding a pooled proportion of 41.4% (95% CI 35.8–47.2). Meanwhile, 34 studies used the ODD test, with a pooled proportion of 39.1% (95% CI 31.5–47.0). There were no significant statistical differences observed between the different detection methods (*p* = 0.8), as shown in Table 1. When restricting the analysis to studies that included patients across all age categories (*n* = 84), the pooled proportion was 40.2% (95% CI 35.7–44.7).

### MRSA proportion categorized by infection source

Stratification based on the source of infection revealed varying MRSA proportion rates, as shown in Table 1. Urinary tract infections exhibited the highest proportion, with a pooled rate of 52.3% (95% CI 37.8–66.6), closely followed by surgical site infections at 49.8% (95% CI 41.7–57.8). Skin and soft tissue infections showed a moderate proportion of 44.2% (95% CI 19.8–70.3), while bloodstream infections had the lowest at 39.0% (95% CI 31.0–47.3). Despite these variations, the differences in across infection sources were not statistically significant (*p* = 0.2).

**Table 1:** Proportion of MRSA in Africa stratified by detection methods and sources of infection

Subgroup	Included studies	Pooled proportion (%)	95% CI (%)	*P*-value for subgroup differences
*Overall*	191	42.2	[38.7–45.6]	NA
*Detection Method*				
*mecA*	52	41.4	[35.8–47.2]	0.8
CDD	94	42.8	[37.2–48.4]	
ODD	34	39.1	[31.5–47.0]	
*Source of infection*				
Bloodstream Infection	30	39.0	[31.0- 47.3]	0.2
Urinary Tract Infection	12	52.3	[37.8–66.6]	
Skin and Soft Tissue Infection	8	44.2	[19.8–70.3]	
Surgical Site Infection	19	49.8	[41.7–57.8]	

*CDD* Cefoxitin disc diffusion, *ODD* Oxacillin disc diffusion, *NA* Not applicable, *CI* Confidence interval

### Proportion of MRSA infection across regions and countries in Africa

**Fig. 3:**
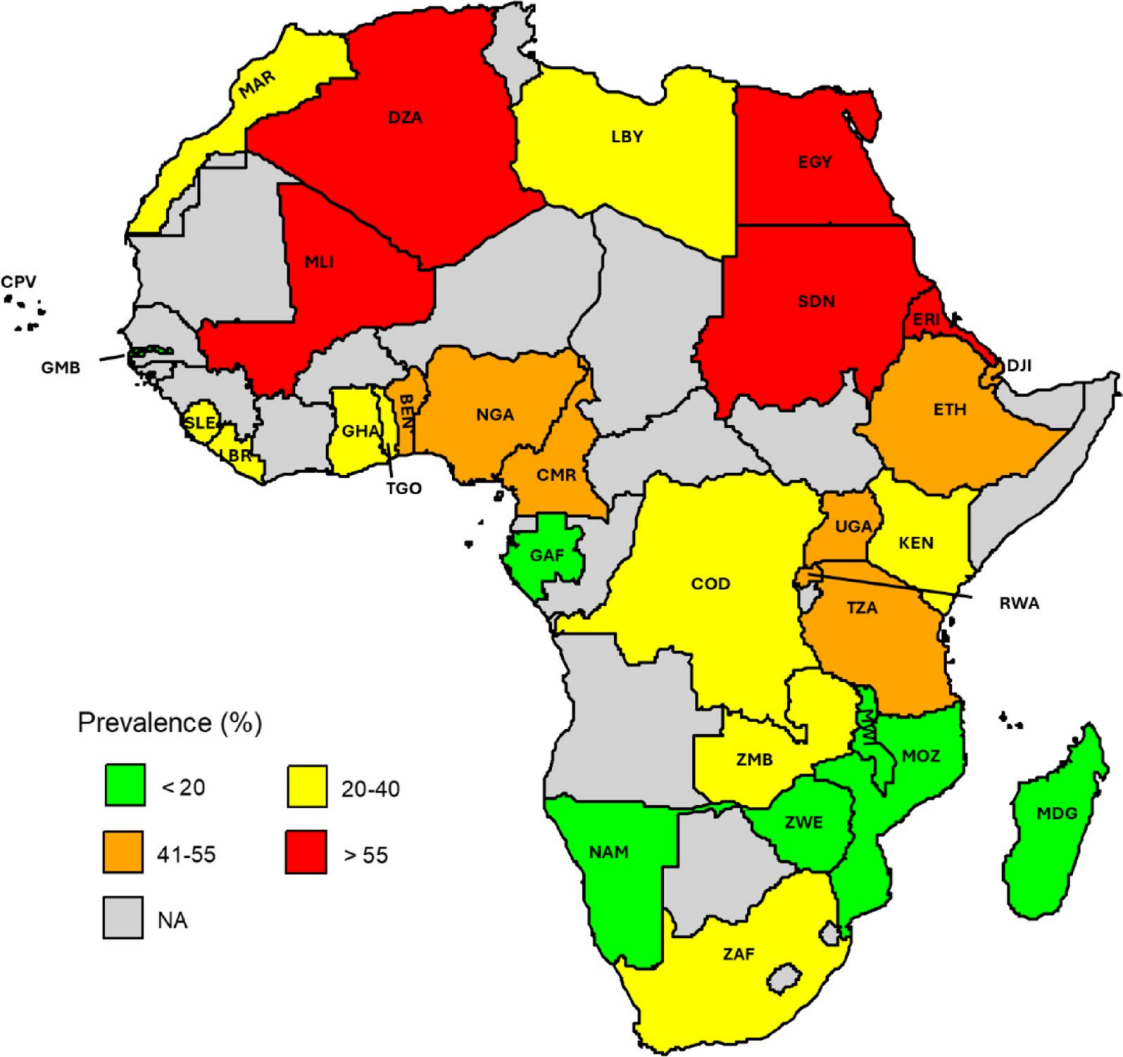
Proportion of MRSA in Africa. Each country is labeled with its respective ISO 3166-1 alpha-3 code. Algeria (DZA), Benin (BEN), Cabo Verde (CPV), Cameroon (CMR), Democratic Republic of the Congo (COD), Djibouti (DJI), Egypt (EGY), Eritrea (ERI), Ethiopia (ETH), Gabon (GAB), Gambia (GMB), Ghana (GHA), Kenya (KEN), Liberia (LBR), Libya (LBY), Madagascar (MDG), Malawi (MWI), Mali (MLI), Mauritius (MUS), Morocco (MAR), Mozambique (MOZ), Namibia (NAM), Nigeria (NGA), Rwanda (RWA), Sierra Leone (SLE), South Africa (ZAF), Sudan (SDN), Tanzania (TZA), Togo (TGO), Uganda (UGA), Zambia (ZMB), and Zimbabwe (ZWE)

As shown in Table 2, based on 54 studies, Northern African countries had the highest pooled proportion of MRSA infection at 56.2% (95% CI 49.3–62.9). Among these, Egypt recorded the highest at 61.8%, followed by Algeria at 60.2% and Sudan at 55.8%, while Morocco reported the lowest at 20.6%. Compared to Northern Africa, Sub-Saharan Africa exhibited a significantly lower pooled proportion of MRSA at 36.7% (95% CI 33.2–40.4); *p* < 0.001. Among the Sub-Saharan regions, Eastern Africa recorded the highest proportion at 39.9% (95% CI 32.2–47.9), followed by Central Africa at 38.3% (95% CI 26.0–51.3), Western Africa at 36.6% (95% CI 31.1–42.8), and Southern Africa at 27.5% (95% CI 21.6–33.6). However, these regional differences were not statistically significant. Within Eastern Africa, Eritrea reported the highest at 71.8% (95% CI 62.5–80.4), while Malawi showed the lowest at 7.0% (95% CI 3.0–12.6). In Central Africa, Cameroon exhibited the highest at 54.0% (95% CI 40.0–67.7), and Gabon recorded the lowest at 8.2% (95% CI 5.0–11.9). In Western Africa, Mali had the highest at 60.3% (95% CI 43.2–76.3), whereas Gambia reported the lowest at 2.4% (95% CI 0.9–4.5). Finally, in Southern Africa, Zambia showed the highest at 39.3% (95% CI 32.1–46.7), while Zimbabwe had the lowest at 9.5% (95% CI 5.6–14.2). The proportion of MRSA across different countries in Africa is illustrated in Fig. 3.

**Table 2:** Proportion of MRSA infection stratified by geographic regions and counties

Country	Included studies	Pooled proportion (%)	95% CI (%)
*a. Northern Africa*			
Overall	54	56.2	[49.3–62.9]
Algeria	11	60.2	[45.0–74.5]
Libya	4	34.1	[25.0–43.9]
Sudan	10	55.8	[41.5–69.6]
Morocco	3	20.6	[9.1–35.1]
Egypt	26	61.8	[51.6–71.5]
*b. Sub-Saharan Africa*			
Overall	137	36.7	[33.2–40.4]
*b.1. Western Africa*			
Overall	53	36.6	[31.1–42.8]
Benin	3	44.4	[19.2–71.2]
Liberia	1	20.7	[13.8–28.6]
Sierra Leone	2	37.7	[11.2–68.8]
Togo	2	21.3	[19.2–23.4]
Cabo Verde	1	21.3	[19.2–23.4]
Ghana	11	26.6	[12.5–43.5]
Mali	3	60.3	[43.2–76.3]
Nigeria	29	41.1	[35.2–47.1]
Gambia	1	2.4	[0.9–4.5]
*b.2. Central Africa*			
Overall	18	38.3	[26.0- 51.3]
Congo DCR	3	39.5	[19.4–61.6]
Gabon	5	8.2	[5.0–11.9]
Cameroon	10	54.0	[40.0–67.7]
*b.3. Eastern Africa*			
Overall	54	39.9	[32.2–47.9]
Djibouti	1	44.6	[35.3–53.9]
Eritrea	2	71.8	[62.5–80.4]
Ethiopia	13	42.3	[32.2–52.8]
Kenya	7	30.7	[11.4–54.3]
Madagascar	1	14.5	[10.0- 20.6]
Mauritius	1	53.3	[27.6–78.3]
Mozambique	4	18.4	[3.6–40.6]
Rwanda	5	52.3	[30.2–73.9]
Tanzania	11	41.1	[31.0–51.6]
Uganda	7	53.4	[41.5–65.2]
Malawi	2	7.0	[3.0–12.6]
*b.4. Southern Africa*			
Overall	17	27.5	[21.6–33.6]
Namibia	1	13.5	[10.9–16.4]
South Africa	10	29.8	[23.5–36.6]
Zambia	4	39.3	[32.1–46.7]
Zimbabwe	2	9.5	[5.6–14.2]

### Meta-analysis of antimicrobial resistance patterns of MRSA to commonly used antibiotic therapies

**Fig. 4:**
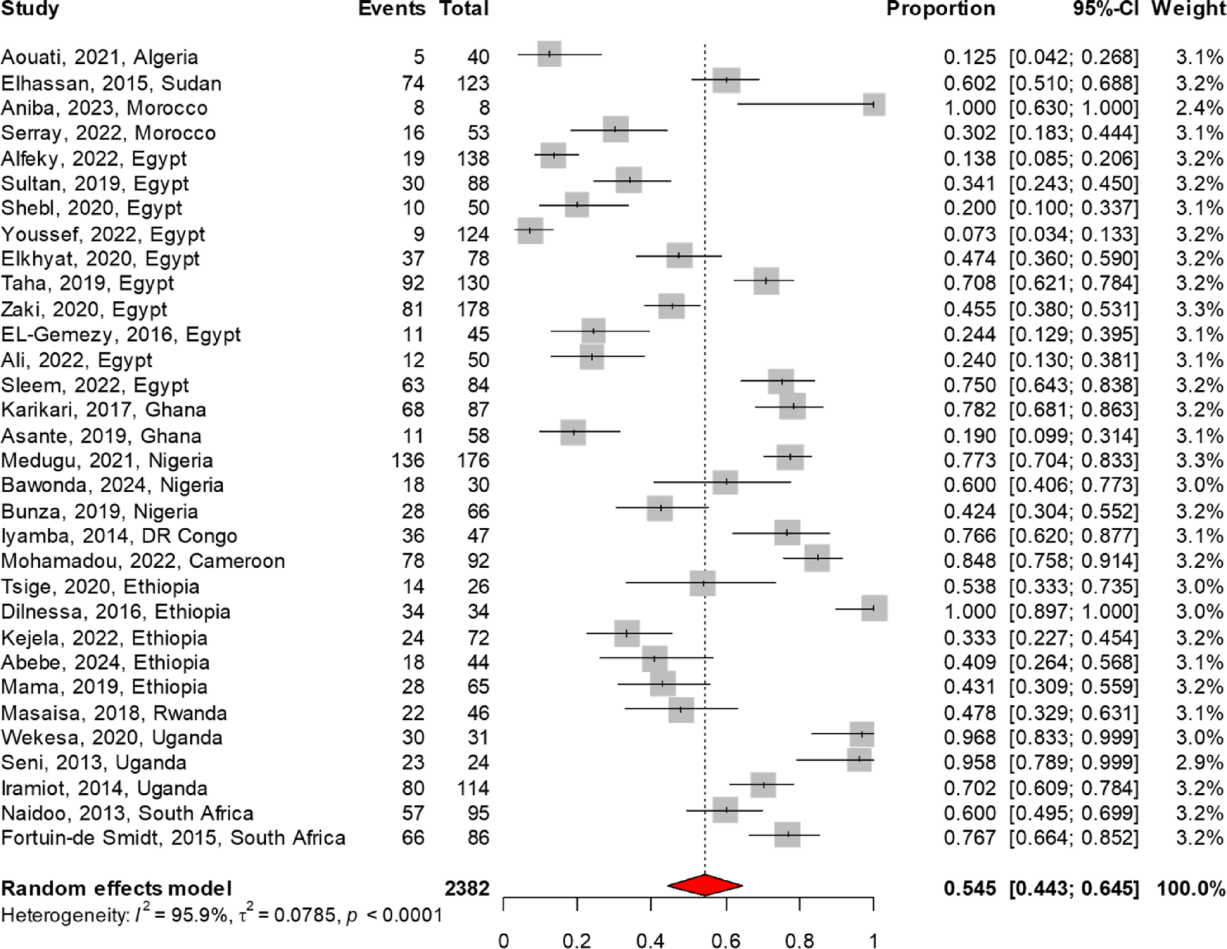
The pooled resistance rate of Trimethoprim-Sulfamethoxazole to MRSA. The meta-analysis, based on a random effects model, estimated resistance at 54.5% (95% CI 44.3–64.5)

**Fig. 5:**
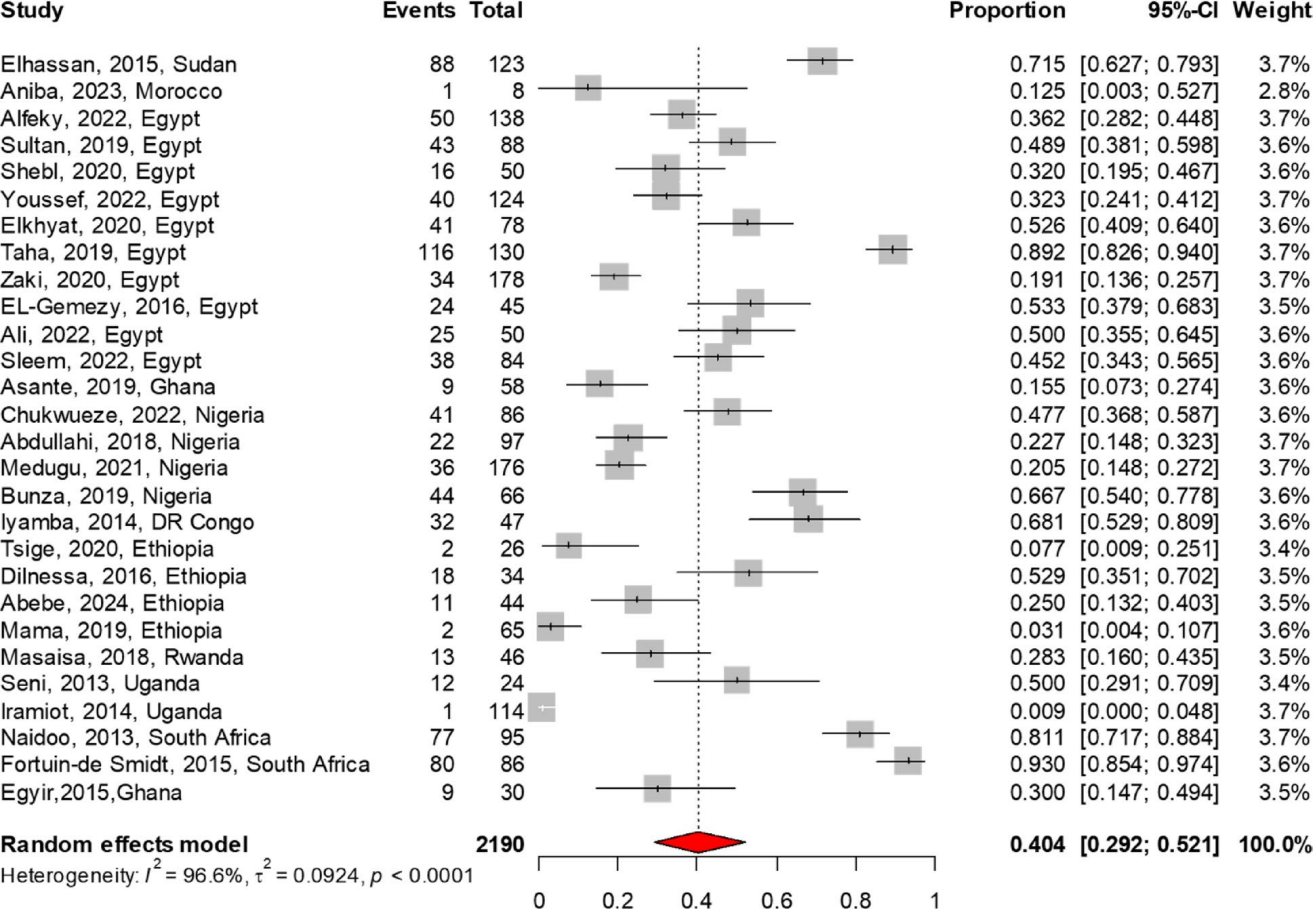
The pooled resistance rate of clindamycin to MRSA. The meta-analysis, based on a random effects model, estimated resistance at 40.4% (95% CI 29.2–52.1)

**Fig. 6:**
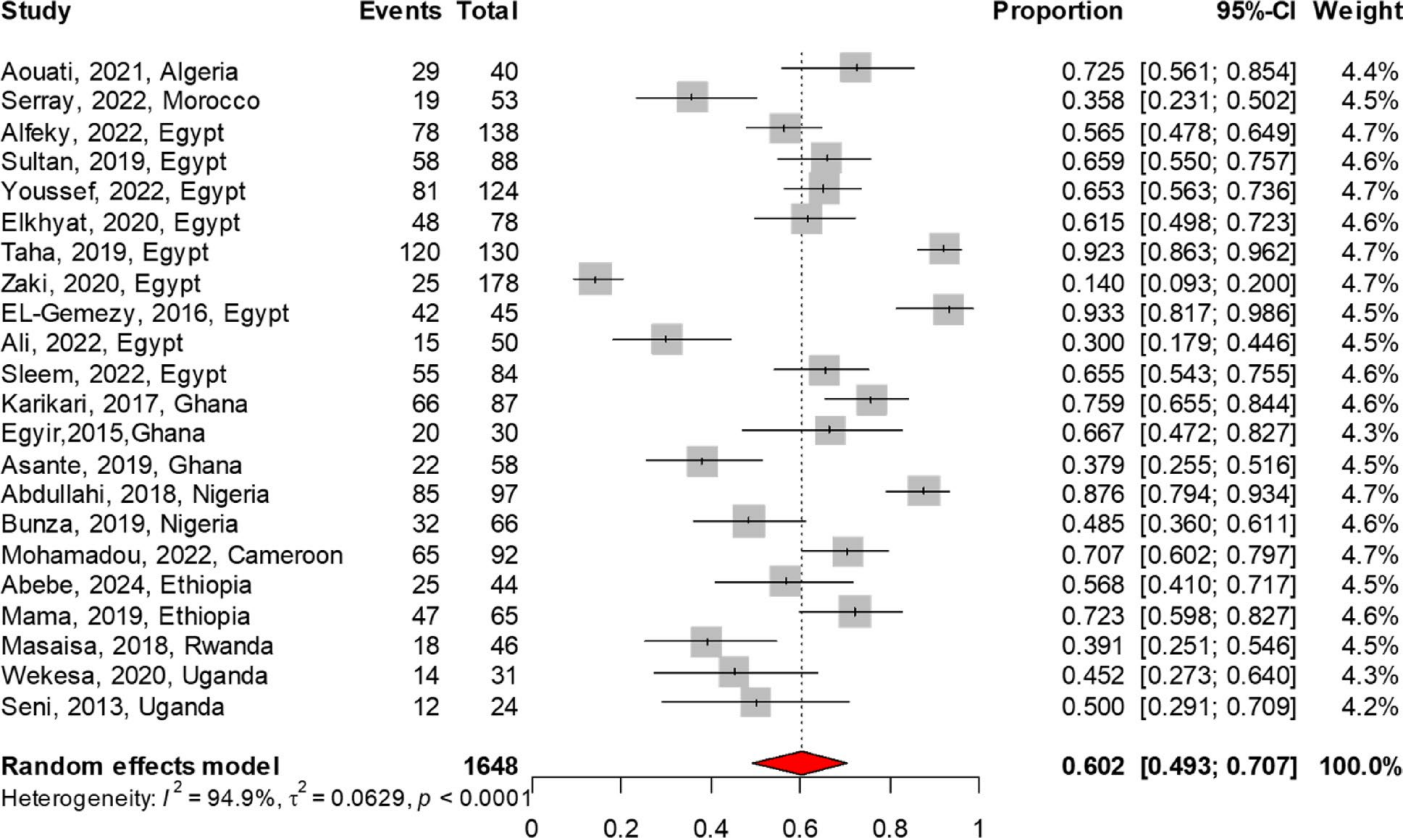
The pooled resistance rate of tetracycline to MRSA. The meta-analysis, based on a random effects model, estimated resistance at 60.2% (95% CI 49.3–70.7)

The MRSA antibiogram profile was reported in 32 studies for TMP-SMX, 29 each for vancomycin and clindamycin, 22 for tetracycline, 19 for linezolid, 11 for rifampin, and 6 for fusidic acid. The meta-analysis findings on AMR patterns of MRSA are summarized in Table 3. Among the antibiotics analyzed, linezolid demonstrated the lowest resistance rate at 3.4% (95% CI 1.3–6.3), followed by vancomycin at 4.7% (95% CI 2.1–8.0). In contrast, higher resistance rates were noted for fusidic acid at 11.6% (95% CI 4.9–20.6), rifampin at 28.4% (95% CI 16.2–42.4), clindamycin at 40.4% (95% CI 29.2–52.1), TMP-SMX at 54.5% (95% CI 44.3–64.5), and tetracycline at 60.2% (95% CI 49.3–70.7). The forest plot of the meta-analysis illustrates the pooled resistance rates of TMP-SMX, clindamycin, and tetracycline to MRSA, as presented in Figs. 4, 5, 6. There were no reports on the resistance patterns of MRSA for the antibiotics telavancin, dalbavancin, oritavancin, or tedizolid. Additionally, there was only one report each on ceftaroline, mupirocin, and daptomycin [43].

**Table 3:** Meta-Analysis of MRSA resistance patterns for commonly used antibiotic therapies

Antibiotic	Included studies	No. of tested MRSA	Proportion (%)	95% CI (%)
Linezolid	19	1461	3.4	[1.3–6.3]
Vancomycin	29	2182	4.7	[2.1- 8.0]
Fusidic Acid	6	349	11.6	[4.9–20.6]
Rifampin	11	977	28.4	[16.2–42.4]
Clindamycin	28	2190	40.4	[29.2–52.1]
Trimethoprim-Sulfamethoxazole	32	2382	54.5	[44.3–64.5]
Tetracycline	22	1648	60.2	[49.3–70.7]

## Discussion

AMR is a pressing global health threat, with Africa facing a disproportionate burden due to widespread antibiotic misuse, weak surveillance systems, and limited ASPs [15, 17, 18, 44]. The proportion of MRSA among *S. aureus* isolates in Africa remains unquantified, with no pooled estimates available. To the best of our knowledge, this is the first meta-analysis to address this gap at the continental level. Our findings reveal a high overall MRSA proportion, with substantial regional variation. This concern is further compounded by alarmingly high resistance rates to multiple antibiotics commonly used in MRSA treatment. These results underscore the urgent need for region-specific ASPs and stronger infection control strategies to combat the escalating MRSA threat in Africa.

The high proportion of MRSA in Africa (42.2%), with most countries reporting rates of 10% or higher, necessitates the consideration of empirical anti-MRSA therapy, particularly for the management of high-risk infections [43]. This widespread burden is likely the result of multiple interrelated factors, chief among them the limited implementation of ASPs across the continent [15]. The effectiveness of such programs is further hindered by systemic challenges, including constrained resources, weak healthcare infrastructure, and a shortage of trained personnel—barriers that collectively limit the capacity to monitor and control antibiotic use effectively [15]. In addition to these structural barriers, the high prevalence of irrational antimicrobial prescribing and inappropriate antibiotic use among hospitalized patients significantly accelerates the spread of MRSA [17, 18]. Compounding this problem is the widespread practice of antibiotic self-medication across Africa, driven by unregulated access, and low public awareness regarding the dangers of antibiotic misuse. For instance, studies have reported varying rates of antibiotic self-medication across the continent: 45.1% in Eritrea [20], 71.3% in Sudan [19], and even higher rates in Egypt (77.7%) [22] and Mali (80.1%) [21]. Addressing these challenges requires the development and implementation of robust ASPs, targeted interventions to optimize antimicrobial use and investments in healthcare systems. Additionally, public campaigns to raise awareness about the dangers of self-medication with antibiotics are essential to mitigate the rising burden of AMR in the region.

The marked regional disparities in MRSA proportion observed in our analysis—such as the significantly higher rates in Northern Africa compared to Sub-Saharan Africa—reflect potential differences in healthcare infrastructure, infection control practices, and antibiotic use patterns. Notably, some Sub-Saharan countries exhibited extreme variation, from very high rates in Eritrea to exceptionally low rates in The Gambia. These findings highlight the need for region-specific interventions and underscore the limitations of generalized approaches. The disparity in MRSA proportion is evident globally. For example, data from the 2021 AMR Surveillance in Europe indicated that MRSA proportion was below 5% in countries such as Norway, Germany, Belgium, and the Netherlands; between 5 and 10% in the United Kingdom; and between 10 and 25% in Spain and France, with no European country reporting rates exceeding 50% [12]. Surveillance data from the China Antimicrobial Surveillance Network (CHINET) for 2022, based on non-duplicated clinical isolates collected from 71 hospitals, reported an MRSA detection rate of 28.7% out of 31,789 tested isolates [45]. In the United States, MRSA proportion was reported at 39.2% in 2021, with 3,980 resistant isolates out of 10,149 tested [9]. In contrast, data from the National Antimicrobial Resistance Surveillance Network in India reported a significantly higher MRSA proportion in blood of 59% in 2022 [11].

Monitoring MRSA resistance patterns is essential for guiding empirical therapy and improving infection management. Our findings revealed pooled resistance rates of 3.4% [95% CI 1.3–6.3%] for linezolid and 4.7% [95% CI 2.1–8.0%] for vancomycin among MRSA isolates. Although these rates are relatively low, they exceed global averages and point to a concerning trend. Notably, linezolid resistance in MRSA remains extremely rare worldwide, making its emergence in the region particularly alarming. Data from the CDC’s National Healthcare Safety Network (NHSN) reported linezolid resistance in only 9 out of 2,491 MRSA isolates (0.4%) and vancomycin resistance in just 5 out of 3,824 isolates (0.1%) in 2021 [46, 47]. The Zyvox^®^ Annual Appraisal of Potency and Spectrum (ZAAPS) program, which monitors linezolid resistance in 32 non-USA countries, found no resistance to linezolid or vancomycin among 1,029 tested MRSA isolates [48]. Similarly, the LEADER program in the USA reported linezolid resistance at 0.1% and no resistance to vancomycin among MRSA isolates [49]. In Europe, linezolid resistance among MRSA blood isolates was notably low, at 0.29% [50]. Similarly, in China, out of 9116 MRSA isolates tested, no resistance to vancomycin or linezolid was detected [45]. This is particularly important as linezolid and vancomycin are first-line treatments for severe MRSA. Resistance to these drugs significantly limits treatment options and complicates the management of severe infections. Future studies should focus on monitoring this concern and investigating the mechanisms of resistance, particularly plasmid-mediated resistance, which facilitates horizontal gene transfer and accelerates dissemination.

The Infectious Diseases Society of America recommends TMP-SMX, tetracycline, and clindamycin as viable treatment options for MRSA infections, particularly skin and soft tissue infections [51]. However, the choice of antibiotic should be guided by local resistance patterns to ensure efficacy. For example, clindamycin can be considered for empiric therapy when local resistance rates are below 10%, as higher resistance rates increase the likelihood of treatment failure [51]. In our study, resistance rates among MRSA isolates were notably high: clindamycin (40.4%, 95% CI 29.2–52.1), TMP-SMX (54.5%, 95% CI 44.3–64.5), and tetracycline (60.2%, 95% CI 49.3–70.7). In contrast, data from the NHSN showed that the percentage of MRSA isolates resistant to TMP-SMX increased from 3.9% in 2012 to 6.5% in 2018 [52]. The CHINET reported MRSA resistance rates of 6.4% for TMP-SMX and 53.6% for clindamycin in 2022 [45]. Interestingly, these findings align more closely with Indian data. According to Indian Network for Surveillance of Antimicrobial Resistance, resistance rates among MRSA isolates for TMP-SMX and clindamycin are 55.6% and 46.6%, respectively [10]. Other studies from India have reported similar ranges, with TMP-SMX resistance rates between 41% and 82.3% and clindamycin resistance rates between 25.6% and 56% [53–57]. Additionally, consistent with our findings, a Malaysian study reported a tetracycline resistance rate of 76.1%, while an Iranian study reported an even higher rate of 84.2% among MRSA isolates [58, 59]. These findings have significant implications for antibiotic selection, highlighting the limited utility of tetracycline, TMP-SMX and clindamycin for MRSA treatment in regions with high resistance rates. Failure to address these resistance patterns may lead to treatment failures, increased morbidity, and higher healthcare costs. These results emphasize the need for updated empiric therapy guidelines, targeted surveillance, ASPs, and a shift toward more effective alternative therapies to ensure optimal patient outcomes.

Overall, our study offers valuable insights into the proportion of MRSA; however, a more comprehensive understanding will require additional data on colonization rates and the molecular epidemiology of MRSA clones. MRSA colonization is a key driver of transmission, and its reduction is vital to lowering infection rates. A recent meta-analysis from Africa reported the highest colonization prevalence among healthcare workers (13.6%) and hospitalized patients (12.9%), with lower rates in community residents (4.1%) and children (4.7%) [60]. MRSA showed high resistance to mupirocin (10.7%), clindamycin (23.6%), and trimethoprim–sulfamethoxazole (38.9%), underscoring the need for ongoing surveillance and robust antimicrobial stewardship efforts. Furthermore, understanding the molecular epidemiology of MRSA clones is essential for identifying outbreak sources, tracking the emergence and spread of high-risk clones, and elucidating the mechanisms underlying antibiotic resistance. Although recent studies have noted an increasing prevalence of clones such as ST1, ST22, and ST152, alongside a decline in ST239/241 in Africa, available data on MRSA clone distribution across the continent remain limited [61]. Expanding molecular surveillance is therefore crucial to inform targeted infection control strategies and antibiotic stewardship efforts.

## Strengths and limitations

This analysis demonstrates notable strengths, including its rigorous methodology, which provides a comprehensive evaluation of MRSA proportion and antibiogram data across Africa. The inclusion of 191 studies with a large sample size of 40,979 isolates significantly enhances the reliability and precision of the findings. The broad geographical representation offers valuable insights into regional proportion patterns, while the fair quality of the included studies and the absence of significant outliers—confirmed through sensitivity analysis—ensure the robustness and consistency of the results. However, several limitations must be acknowledged. First, only 32 out of 54 African countries were represented in the included studies, with some countries significantly underrepresented. For example, Namibia contributed only one study and Malawi two, limiting the generalizability of the findings. Second, the analysis demonstrated significant heterogeneity (I^2^ = 96.3%), a common occurrence in prevalence meta-analyses due to the intrinsic variability of proportional data [62]. Third, although we stratified MRSA proportion by infection type, further stratification by region or country was not feasible due to the limited number of studies available for each infection type within individual countries. Another limitation of this study is that it was not pre-registered. However, we developed a protocol and adhered to it without modifications in the pre-specified analysis, ensuring methodological rigor by strictly following the PRISMA guidelines. Additionally, a significant challenge was the inability to stratify MRSA infections by mode of acquisition (community-acquired vs. hospital-acquired), primarily due to inconsistent and non-standardized definitions across the included studies. Some studies identified hospital-acquired infections based on the timing of onset (e.g., more than 48–72 h after hospital admission), while others classified cases using *SCCmec* typing or based solely on the patient’s care setting (inpatient vs. outpatient). These varying criteria made it difficult to apply a consistent classification across studies, thereby limiting our ability to conduct meaningful subgroup analysis based on infection acquisition. These inconsistencies hindered a more detailed and rigorous meta-analysis. In light of these limitations, there is a clear need for additional, well-designed research, particularly in underrepresented regions and countries with no available data. Addressing these gaps will be essential to improve the understanding of MRSA proportion and generate more comprehensive estimates across Africa.

## Conclusion

The proportion of MRSA in Africa remains high at 42.2%. Although resistance rates to linezolid and vancomycin are relatively low, they exceed global averages, indicating a worrisome trend. This concern is further compounded by exceptionally high resistance rates to other antibiotics commonly used for MRSA treatment, significantly reducing their efficacy. Future research should focus on monitoring resistance patterns and investigating mechanisms such as plasmid-mediated resistance, which facilitate rapid dissemination. These findings emphasize the urgent need for targeted interventions, including strengthened ASPs and robust surveillance systems, to mitigate the growing threat of MRSA.

## Supplementary Information

Below is the link to the electronic supplementary material.

Supplementary Material 1

## Data Availability

All data generated and analyzed throughout this study were included either in this article or its supplementary information file.

## Abbreviations

MRSA: Methicillin-resistant *Staphylococcus aureus*
MSSA: Methicillin-susceptible *Staphylococcus aureus*
*S. aureus*: *Staphylococcus aureus*
PBP2a: Penicillin-binding Protein 2a
*SCCmec*: Staphylococcal cassette chromosome mec
ASPs: Antimicrobial Stewardship Programs
CDD: Cefoxitin disc diffusion
ODD: Oxacillin disc diffusion
TMP-SMX: Trimethoprim/sulfamethoxazole
AMR: Antimicrobial resistance
PRISMA: Preferred reporting items for systemic review and meta-analysis
MIC: Minimum inhibitory concentration
RLAT: Rapid latex agglutination test
NHSN: National Healthcare Safety Network
ZAAPS: Zyvox Annual appraisal of potency and spectrum
LEADER: Linezolid experience and accurate determination of resistance
CHINET: The China antimicrobial surveillance network
