# Supplementary material for: Proportion and antibiogram of methicillin-resistant Staphylococcus aureus (MRSA) in Africa: a systematic review and meta-analysis
Source: Antimicrob Resist Infect Control. 2026 Jan 21;15:13. doi: 10.1186/s13756-025-01687-3 (PMC12849242; doi:10.1186/s13756-025-01687-3)
Supplement: Supplementary file 1 — Supplementary Material 1 [file 13756_2025_1687_MOESM1_ESM.docx]

**Additional file**

**Prevalence and Antibiogram of Methicillin-resistant *Staphylococcus aureus* (MRSA) in Africa: A Systematic Review and Meta-Analysis**

**Table S1**: The detailed search strategy

| **Category** | **Search Terms** |
| --- | --- |
| **MRSA Terms** | "Methicillin-resistant Staphylococcus aureus", "Methicillin resistant Staphylococcus aureus", MRSA |
| **Clinical Terms** | Patient*, Outpatient*, Inpatient*, Individual*, Human*, Neonate*, Pediatric*, Child*, Infant*, Newborn*, Adult*, Adolescent*, Elderly, "Older Individual*", "Older Adult*", "Clinical Specimen*", "Clinical Sample*", "Clinical Isolate*" |
| **African Countries** | Africa*, Algeria, Angola, Benin, Botswana, "Burkina Faso", Burundi, "Cabo Verde", Cameroon, "Central African Republic", Chad, Comoros, Congo, "Democratic Republic of the Congo", Djibouti, Egypt, "Equatorial Guinea", Eritrea, Eswatini, Ethiopia, Gabon, Gambia, Ghana, Guinea, "Guinea-Bissau", "Ivory Coast", Kenya, Lesotho, Liberia, Libya, Madagascar, Malawi, Mali, Mauritania, Mauritius, Morocco, Mozambique, Namibia, Niger, Nigeria, Rwanda, "São Tomé and Príncipe", Senegal, Seychelles, "Sierra Leone", Somalia, "South Africa", "South Sudan", Sudan, Tanzania, Togo, Tunisia, Uganda, Zambia, Zimbabwe |

**Table S2**: The Detailed search strategy for Scopus and PubMed Databases

| Database | Search Strategy |
| --- | --- |
| Scopus | ( TITLE-ABS-KEY ( "Methicillin-resistant Staphylococcus aureus" OR "Methicillin resistant Staphylococcus aureus" OR MRSA ) )  AND ( TITLE-ABS-KEY ( Patient* OR Outpatient* OR Inpatient* OR Individual* OR Human* OR Neonate* OR Pediatric* OR Child* OR Infant* OR Newborn* OR Adult* OR Adolescent* OR Elderly OR "Older Individual*" OR "Older Adult*" OR "Clinical Specimen*" OR "Clinical Sample*" OR "Clinical Isolate*" ) )  AND ( TITLE-ABS-KEY ( Africa* OR Algeria OR Angola OR Benin OR Botswana OR "Burkina Faso" OR Burundi OR "Cabo Verde" OR Cameroon OR "Central African Republic" OR Chad OR Comoros OR Congo OR "Democratic Republic of the Congo" OR Djibouti OR Egypt OR "Equatorial Guinea" OR Eritrea OR Eswatini OR Ethiopia OR Gabon OR Gambia OR Ghana OR Guinea OR "Guinea-Bissau" OR "Ivory Coast" OR Kenya OR Lesotho OR Liberia OR Libya OR Madagascar OR Malawi OR Mali OR Mauritania OR Mauritius OR Morocco OR Mozambique OR Namibia OR Niger OR Nigeria OR Rwanda OR "São Tomé and Príncipe" OR Senegal OR Seychelles OR "Sierra Leone" OR Somalia OR "South Africa" OR "South Sudan" OR Sudan OR Tanzania OR Togo OR Tunisia OR Uganda OR Zambia OR Zimbabwe ) )  AND (PUBYEAR > 2012 AND PUBYEAR < 2025) |
| PubMed | (("Methicillin-resistant Staphylococcus aureus"[Title/Abstract] OR "Methicillin resistant Staphylococcus aureus"[Title/Abstract] OR MRSA[Title/Abstract])  AND  (Patient*[Title/Abstract] OR Outpatient*[Title/Abstract] OR Inpatient*[Title/Abstract] OR Individual*[Title/Abstract] OR Human*[Title/Abstract] OR Neonate*[Title/Abstract] OR Pediatric*[Title/Abstract] OR Child*[Title/Abstract] OR Infant*[Title/Abstract] OR Newborn*[Title/Abstract] OR Adult*[Title/Abstract] OR Adolescent*[Title/Abstract] OR Elderly[Title/Abstract] OR "Older Individual*"[Title/Abstract] OR "Older Adult*"[Title/Abstract] OR "Clinical Specimen*"[Title/Abstract] OR "Clinical Sample*"[Title/Abstract] OR "Clinical Isolate*"[Title/Abstract])  AND  (Africa*[Title/Abstract] OR Algeria[Title/Abstract] OR Angola[Title/Abstract] OR Benin[Title/Abstract] OR Botswana[Title/Abstract] OR "Burkina Faso"[Title/Abstract] OR Burundi[Title/Abstract] OR "Cabo Verde"[Title/Abstract] OR Cameroon[Title/Abstract] OR "Central African Republic"[Title/Abstract] OR Chad[Title/Abstract] OR Comoros[Title/Abstract] OR Congo[Title/Abstract] OR "Democratic Republic of the Congo"[Title/Abstract] OR Djibouti[Title/Abstract] OR Egypt[Title/Abstract] OR "Equatorial Guinea"[Title/Abstract] OR Eritrea[Title/Abstract] OR Eswatini[Title/Abstract] OR Ethiopia[Title/Abstract] OR Gabon[Title/Abstract] OR Gambia[Title/Abstract] OR Ghana[Title/Abstract] OR Guinea[Title/Abstract] OR "Guinea-Bissau"[Title/Abstract] OR "Ivory Coast"[Title/Abstract] OR Kenya[Title/Abstract] OR Lesotho[Title/Abstract] OR Liberia[Title/Abstract] OR Libya[Title/Abstract] OR Madagascar[Title/Abstract] OR Malawi[Title/Abstract] OR Mali[Title/Abstract] OR Mauritania[Title/Abstract] OR Mauritius[Title/Abstract] OR Morocco[Title/Abstract] OR Mozambique[Title/Abstract] OR Namibia[Title/Abstract] OR Niger[Title/Abstract] OR Nigeria[Title/Abstract] OR Rwanda[Title/Abstract] OR "São Tomé and Príncipe"[Title/Abstract] OR Senegal[Title/Abstract] OR Seychelles[Title/Abstract] OR "Sierra Leone"[Title/Abstract] OR Somalia[Title/Abstract] OR "South Africa"[Title/Abstract] OR "South Sudan"[Title/Abstract] OR Sudan[Title/Abstract] OR Tanzania[Title/Abstract] OR Togo[Title/Abstract] OR Tunisia[Title/Abstract] OR Uganda[Title/Abstract] OR Zambia[Title/Abstract] OR Zimbabwe[Title/Abstract])  AND  ("2013/01/01"[Publication Date] : "2024/12/31"[Publication Date])) |

**Table S3: The 27-Item Checklist of the PRISMA Statement**

| Section/topic | Item No | Checklist item | Reported on page No |
| --- | --- | --- | --- |
| Title | | | |
| Title | 1 | Identify the report as a systematic review, meta-analysis, or both | 1 |
| Abstract | | | |
| Structured summary | 2 | Provide a structured summary including, as applicable, background, objectives, data sources, study eligibility criteria, participants, interventions, study appraisal and synthesis methods, results, limitations, conclusions and implications of key findings, systematic review registration number | 2,3 |
| Introduction | | | |
| Rationale | 3 | Describe the rationale for the review in the context of what is already known | 5 |
| Objectives | 4 | Provide an explicit statement of questions being addressed with reference to participants, interventions, comparisons, outcomes, and study design (PICOS) | 5 |
| Methods | | | |
| Protocol and registration | 5 | Indicate if a review protocol exists, if and where it can be accessed (such as web address), and, if available, provide registration information including registration number | 20 |
| Eligibility criteria | 6 | Specify study characteristics (such as PICOS, length of follow-up) and report characteristics (such as years considered, language, publication status) used as criteria for eligibility, giving rationale | 6 |
| Information sources | 7 | Describe all information sources (such as databases with dates of coverage, contact with study authors to identify additional studies) in the search and date last searched | 5 |
| Search | 8 | Present full electronic search strategy for at least one database, including any limits used, such that it could be repeated | Tables S1 and S2 |
| Study selection | 9 | State the process for selecting studies (that is, screening, eligibility, included in systematic review, and, if applicable, included in the meta-analysis) | 6 |
| Data collection process | 10 | Describe method of data extraction from reports (such as piloted forms, independently, in duplicate) and any processes for obtaining and confirming data from investigators | 6&7 |
| Data items | 11 | List and define all variables for which data were sought (such as PICOS, funding sources) and any assumptions and simplifications made | 6 |
| Risk of bias in individual studies | 12 | Describe methods used for assessing risk of bias of individual studies (including specification of whether this was done at the study or outcome level), and how this information is to be used in any data synthesis | 7  **Table S4 and S5.** |
| Summary measures | 13 | State the principal summary measures (such as risk ratio, difference in means). | 8 |
| Synthesis of results | 14 | Describe the methods of handling data and combining results of studies, if done, including measures of consistency (such as I^2^ statistic) for each meta-analysis | 8 |
| Risk of bias across studies | 15 | Specify any assessment of risk of bias that may affect the cumulative evidence (such as publication bias, selective reporting within studies) | Not applicable |
| Additional analyses | 16 | Describe methods of additional analyses (such as sensitivity or subgroup analyses, meta-regression), if done, indicating which were pre-specified | 8 |
| Results | | | |
| Study selection | 17 | Give numbers of studies screened, assessed for eligibility, and included in the review, with reasons for exclusions at each stage, ideally with a flow diagram | 7  Fig. 1 |
| Study characteristics | 18 | For each study, present characteristics for which data were extracted (such as study size, PICOS, follow-up period) and provide the citations | 8 and  Table 1 |
| Risk of bias within studies | 19 | Present data on risk of bias of each study and, if available, any outcome-level assessment (see item 12). | Table S4 and S5 |
| Results of individual studies | 20 | For all outcomes considered (benefits or harms), present for each study (a) simple summary data for each intervention group and (b) effect estimates and confidence intervals, ideally with a forest plot | Tables 1-3 and Figs 4-6 |
| Synthesis of results | 21 | Present results of each meta-analysis done, including confidence intervals and measures of consistency | Tables 1-3 and Figs 4-6 |
| Risk of bias across studies | 22 | Present results of any assessment of risk of bias across studies (see item 15) | Not applicable |
| Additional analysis | 23 | Give results of additional analyses, if done (such as sensitivity or subgroup analyses, meta-regression) (see item 16) | 9-15, Tables 1-3 and Figs 4-6 |
| Discussion | | | |
| Summary of evidence | 24 | Summarize the main findings including the strength of evidence for each main outcome; consider their relevance to key groups (such as health care providers, users, and policy makers) | 15 |
| Limitations | 25 | Discuss limitations at study and outcome level (such as risk of bias), and at review level (such as incomplete retrieval of identified research, reporting bias) | 19&20 |
| Conclusions | 26 | Provide a general interpretation of the results in the context of other evidence, and implications for future research | 20&21 |
| Funding | | | |
| Funding | 27 | Describe sources of funding for the systematic review and other support (such as supply of data) and role of funders for the systematic review | 22 |

**Table S4**: The checklist items for Joanna Briggs's critical appraisal tool for prevalence studies

|  | Yes | No | Unclear | Not applicable |
| --- | --- | --- | --- | --- |
| 1. Was the sample frame appropriate to address the target population? | □ | □ | □ | □ |
| 1. Were study participants sampled in an appropriate way? | □ | □ | □ | □ |
| 1. Was the sample size adequate? | □ | □ | □ | □ |
| 1. Were the study subjects and the setting described in detail? | □ | □ | □ | □ |
| 1. Was the data analysis conducted with sufficient coverage of the identified sample? | □ | □ | □ | □ |
| 1. Were valid methods used for the identification of the condition? | □ | □ | □ | □ |
| 1. Was the condition measured in a standard, reliable way for all participants? | □ | □ | □ | □ |
| 1. Was there appropriate statistical analysis? | □ | □ | □ | □ |
| 1. Was the response rate adequate, and if not, was the low response rate managed appropriately? | □ | □ | □ | □ |

**Table S5**: characteristics of the included articles

| **References** | **last name of the first author** | **publication time** | **Study time** | **Country** | **Africa geographical regions** | **Age Group** | **Type of infection** | **Infection Source** | **Sample Collection Setting** | **Total *S. aureus*** | **Total MRSA** | **Diagnostic method** | **Specimen** | **Evaluation (Out of 8)** |
| --- | --- | --- | --- | --- | --- | --- | --- | --- | --- | --- | --- | --- | --- | --- |
| [1] | Djahmi | 2013 | 1 April 2011 - 30 March 2012 | Algeria | Northern | Adult and Older Adult, 23–83 | DFI | NA | Hospital | 85 | 73 | CDD | Wound | 5 |
| [2] | Aouati | 2021 | January 2012 – December 2013 | Algeria | Northern | All Ages, 2–84 | Various | Hospital-acquired (SCCmec typing) | Hospital | 50 | 40 | mecA | Blood, tracheal drain | 7 |
| [3] | Mesbahi | 2021 | June 2015 – April 2018 | Algeria | Northern | All Ages, 2–84 | Burn Wound | NA | Hospital | 70 | 52 | CDD | Wound | 5 |
| [4] | Rahima | 2015 | 2010–2012 | Algeria | Northern | Adult,18–65 | Various | Community | Private lab | 150 | 67 | ODD | Pus, Urine, vaginal | 7 |
| [5] | Achek | 2018 | Not specified | Algeria | Northern | NA | Various | Both | Hospital | 39 | 18 | CDD | Pus, sperm, Urine, vaginal, Wound, catheter tips and secretions | 6 |
| [6] | Aidaoui | 2022 | August 2019 – August 2020 | Algeria | Northern | All Ages | Various | Both | Hospital | 125 | 80 | CDD | Pus, Blood cultures | 7 |
| [7] | Rahmani | 2021 | January – October 2020 | Algeria | Northern | All Ages, ≤ 14–≥ 60 | Various | Community | Private lab | 43 | 11 | CDD | Pus, Urine, skin, Blood, vaginal. | 7 |
| [8] | Aiza | 2023 | 2016–2020 | Algeria | Northern | All Ages, 0.08–90 | Wound | NA | Hospital | 77 | 24 | CDD | Pus | 5 |
| [9] | Bouharkat | 2020 | March 2016 – June 2018 | Algeria | Northern | Older Adult, 63–64 | DFI | Inpatients | Hospital | 60 | 11 | CDD | Wound | 7 |
| [10] | Hecini-Hannachi | 2016 | 2011–2015 | Algeria | Northern | NA | Various | Inpatients | Hospital | 352 | 232 | CDD | Blood, various samples | 7 |
| [11] | Benyagoub | 2024 | Not specified | Algeria | Northern | Adult | UTI | NA | Hospital | 50 | 50 | CDD | Urine samples | 5 |
| [12] | Wareg | 2014 | October 2009 – November 2010 | Libya | Northern | Adult, 30–49 (Most of cases) | Various | Inpatients and Outpatients | Hospital | 399 | 162 | CDD | Blood, Wound, Pus, nasal swabs | 8 |
| [13] | Ahmad | 2018 | January 2015 – January 2017 | Libya | Northern | All Ages | Various | Inpatients and Outpatients | Hospital | 43 | 7 | CDD | Wound, Pus, oropharyngeal swabs | 7 |
| [14] | Aetrugh | 2017 | June 2013 – June 2014 | Libya | Northern | NA | Various | Inpatients and Outpatients | Hospital | 156 | 62 | CDD | Blood, Pus, nasal swabs | 6 |
| [15] | Eldukali | 2022 | January 2017 – December 2019 | Libya | Northern | Adult, Older Adult, 18–88 | DFI | Inpatients | Hospital | 20 | 7 | CDD | Pus, Wound swabs | 7 |
| [16] | Elhassan | 2015 | April 2013 – October 2014 | Sudan | Northern | Adult, Older Adult, 18–88 | Various | NA | hospitals | 200 | 111 | mecA | Wound, Urine, nasal, ear swabs | 5 |
| [17] | Abdalla | 2019 | October 2017 – May 2018 | Sudan | Northern | NA | SSI | Inpatients | Hospital | 94 | 42 | CDD | Wound | 6 |
| [18] | Hamdan | 2021 | November 2020 – January 2021 | Sudan | Northern | All Ages | Various | Both | Hospital | 300 | 185 | CDD | Respiratory tract, Blood samples | 8 |
| [19][19] | Elimam | 2014 | April 2010 – May 2011 | Sudan | Northern | Pediatric, Adult, 11–49 (Most of cases) | Wound, UTIs | Hospital | Hospital | 49 | 26 | mecA | Wound, Urine, ear, nasal swabs | 7 |
| [20] | Babiker | 2018 | March – July 2016 | Sudan | Northern | Neonate, 0–28 days | Sepsis | Inpatients | Hospital | 45 | 18 | ODD | Blood | 7 |
| [21] | Moglad | 2022 | October 2019 – March 2020 | Sudan | Northern | NA | Various | Inpatients and Outpatients​. | Hospital | 37 | 25 | CDD | Pus, Blood, Urine | 6 |
| [22] | Abdelaziz | 2019 | April 2017 – April 2018 | Sudan | Northern | Neonate, 0–28 days | Sepsis | Inpatients | Hospital | 71 | 68 | ODD | Blood | 7 |
| [23] | Osman | 2018 | 2013–2015 | Sudan | Northern | NA | Various | Both (CDC definition) | Hospital | 210 | 96 | mecA | Wound and Blood | 6 |
| [24] | Ibrahim | 2017 | March 2013–December 2014 | Sudan | Northern | All Ages, 5–80 | Wound | Inpatients and Outpatients​. | Hospital | 232 | 45 | CDD | Wound swabs | 7 |
| [25] | Alhady | 2021 | April–August 2017 | Sudan | Northern | NA | Various | NA | Hospital | 53 | 36 | ODD | Pus, Urine, body fluids​. | 5 |
| [26] | Aniba | 2023 | January 2017 – December 2020 | Morocco | Northern | All Ages, 0.08–97 | UTI | Community | Private lab and Hospital | 18 | 8 | mecA | Urine | 5 |
| [27] | Serray | 2022 | December 2010 – May 2014 | Morocco | Northern | Pediatric, 0–15 | Various | NA | Hospital | 259 | 53 | mecA | Blood, catheters, respiratory samples | 5 |
| [28] | Mourabit | 2014 | August 2008 to August 2011 | Morocco | Northern | All Ages, 15–90 | Various | Both (48-hour criterion) | Hospital | 80 | 8 | mecA | Wound, Blood | 7 |
| [29] | Elbargisy | 2020 | January – December 2019 | Egypt | Northern | NA | Various | NA | Hospital | 123 | 64 | mecA | Pus, Sputum, Urine | 5 |
| [30] | Sonbol | 2022 | Not specified | Egypt | Northern | NA | Ear infections | NA | Hospital | 108 | 68 | ORSAB | Ear swabs | 5 |
| [31] | AbdEl-Mongy | 2018 | Not specified | Egypt | Northern | Neonate | Sepsis | Hospital-acquired | Hospital | 42 | 34 | mecA | Blood cultures | 7 |
| [32] | Hashem | 2013 | Not specified | Egypt | Northern | NA | Various | NA | Hospital | 94 | 45 | ODD | Blood, Wound swabs | 5 |
| [33] | Alfeky | 2022 | September 2017 – December 2018 | Egypt | Northern | All Ages, 0–>60 | Various | NA | Hospital | 170 | 138 | mecA | Wound, Blood, Sputum, Urine | 5 |
| [34] | Omar | 2014 | Not specified | Egypt | Northern | All Ages, 15–70 | Various | Inpatients | Hospital | 100 | 75 | mecA | Sputum, tracheal aspirates, Pus, Blood, and Urine | 7 |
| [35] | El-baz | 2017 | March – November 2014 | Egypt | Northern | NA | Various | Both (SCCmec typing) | Hospital | 136 | 85 | CDD | Urine, Wound, abscesses, Sputum, vaginal smear, nasal discharge and boils | 6 |
| [36] | Kadry | 2016 | _ | Egypt | Northern | NA | Various | NA | Hospital | 117 | 114 | mecA | Wound Blood, Sputum, ear, Urine | 5 |
| [37] | Sultan | 2019 | January – October 2018 | Egypt | Northern | Pediatric, 6.1 ± 2.8 | Various | Inpatients and Outpatients​. | Hospital | 180 | 88 | mecA | Blood, Urine, Sputum, Wound | 7 |
| [38] | Barakat | 2016 | July 2013 to January 2015 | Egypt | Northern | NA | SSI | NA | Hospital | 161 | 73 | ODD | Pus, Wound swabs | 5 |
| [39] | Shebl | 2020 | April – December 2018 | Egypt | Northern | Adult, Older Adult, 18–75 | Various | Inpatients | Hospital | 163 | 50 | CDD | Blood, Pus, Sputum, surgical swabs | 7 |
| [40] | El-Sweify | 2021 | Through ten months | Egypt | Northern | NA | Various | Hospital-acquired (48-hour criterion) | Hospital | 120 | 90 | mecA | Blood, Sputum, BAL, bone biopsy, CSF | 6 |
| [41] | kishk | 2019 | May 2018 – May 2019 | Egypt | Northern | All Ages | BSI | Inpatients | Hospital | 100 | 55 | mecA | Blood | 7 |
| [42] | Mashaly | 2018 | December 2015 – November 2016 | Egypt | Northern | NA | Various | NA | Hospital | 100 | 92 | CDD | Blood, Urine, Wound, nasopharyngeal swabs, Pus | 5 |
| [43] | Youssef | 2022 | 2018–2019. | Egypt | Northern | All Ages, 0–60 | Various | Inpatients | Hospital | 200 | 124 | mecA | Wound, Sputum, Blood, ascites fluid, Urine, ear swabs, Wound | 7 |
| [44] | Shrief | 2019 | January 2017 till March 2018. | Egypt | Northern | NA | SSI | Hospital-acquired | Hospital | 170 | 90 | CDD | Wound | 6 |
| [45] | Elkhyat | 2020 | January2018 to December 2019. | Egypt | Northern | Pediatric, Adult, 22.3 ± 16.2 | Wound | NA | Hospital | 84 | 78 | mecA | Wound | 5 |
| [46] | Taha | 2019 | 2015 | Egypt | Northern | Adult, 40–50 | Various | NA | Hospital | 642 | 129 | mecA | Blood, Urine, respiratory aspirate | 8 |
| [47] | Abdelraheem | 2021 | April 2019 to December 2019 | Egypt | Northern | NA | Wound | NA | Hospital | 116 | 95 | MICs of oxacillin | Wound | 5 |
| [48] | Zaki | 2020 | January 2015 – March 2018 | Egypt | Northern | Pediatric, 1.7 – 5.4 | Sepsis | Hospital-acquired | Hospital | 250 | 178 | ODD | Blood, Wound, central venous lines | 7 |
| [49] | EL-Gemezy | 2016 | _ | Egypt | Northern | NA | Various | Inpatients | Hospital | 100 | 45 | CDD | Blood | 6 |
| [50] | Fahim | 2021 | 2018- 2019 | Egypt | Northern | NA | Various | Inpatients | Hospital | 69 | 51 | CDD | Urine, Wound, Blood | 6 |
| [51] | Ali | 2022 | July 2019 to December 2022 | Egypt | Northern | All Ages, 10–76 | SSI | Hospital-acquired | Hospital | 73 | 50 | CDD | Wound | 7 |
| [52] | Sleem | 2022 | February 2021 to May 2022. | Egypt | Northern | Pediatric, Adult, 33.6 ± 12 | Various | Inpatients | Hospital | 152 | 84 | CDD | Blood, Pus, Urine, Sputum, Wound | 7 |
| [53] | Ibrahim | 2020 | January 2016 and December 2017 | Egypt | Northern | NA | Various | NA | Hospital | 258 | 70 | ORSAB | Urine, Pus, throat, Blood, seminal fluid, prostatic fluid, Sputum | 5 |
| [54] | Hassan | 2017 | _ | Egypt | Northern | Neonate, Pediatric | Various | Hospital-acquired (48-hour criterion) | Hospital | 90 | 18 | CDD | Blood, Urine, endotracheal aspirate, Wound, CSF | 7 |
| [55] | Yehouenou | 2020 | 2019-2020 | Benin | Western | All Ages, Median = 29 (IQR 24–34) | SSI | Hospital-acquired | Hospital | 42 | 32 | CDD | Wound | 7 |
| [56] | Lai (a) | 2018 | 2004-2016 | Benin | Western | All Ages,1–70 | Wound | NA | Other | 191 | 66 | ODD | Wound | 5 |
| [56] | Lai (b) | 2018 | 2004-2016 | Congo | Central | All Ages,1–70 | Wound | NA | Other | 113 | 36 | ODD | Wound | 5 |
| [56] | Lai (c) | 2018 | 2004-2016 | Liberia | Western | All Ages,1–70 | Wound | NA | Other | 116 | 24 | ODD | Wound | 5 |
| [56] | Lai (d) | 2018 | 2004-2016 | Madagascar | Eastern | All Ages,1–70 | Wound | NA | Other | 172 | 25 | ODD | Wound | 5 |
| [56] | Lai (e) | 2018 | 2004-2016 | Sierra Leone | Western | All Ages,1–70 | Wound | NA | Other | 166 | 40 | ODD | Wound | 5 |
| [56] | Lai (f) | 2018 | 2004-2016 | Togo | Western | All Ages,1–70 | Wound | NA | Other | 98 | 14 | ODD | Wound | 5 |
| [57] | Ombelet | 2022 | 2017-2020 | Benin | Western | All Ages | BSI | Both (48-hour criterion) | Hospital | 46 | 11 | ODD | Blood | 7 |
| [58] | Monteiroa | 2021 | 2018-2020 | Cabo Verde | Western | NA | Various | NA | Hospital | 1456 | 310 | ODD | Blood, Wound | 6 |
| [59] | Egyir (a) | 2014 | 2010-2012 | Ghana | Western | NA | Various | NA | Hospital | 308 | 9 | mecA | Pus, Blood | 6 |
| [60] | Anafo | 2021 | 2020 | Ghana | Western | All Ages,13–80 | DFI | NA | Hospital | 19 | 6 | mecA | Wound | 5 |
| [61] | Karikari | 2017 | 2006-2007 | Ghana | Western | All Ages, <1–60 | Various | Outpatients and Inpatients | Hospital | 250 | 87 | Oxacillin E-test | Blood, Pus, Wound | 7 |
| [62] | Opintan | 2017 | 2014 | Ghana | Western | All Ages, <1–>50 | BSI | NA | Hospital | 34 | 0 | mecA | Blood | 5 |
| [63] | Bediako-Bowan | 2020 | 2017-2019 | Ghana | Western | Mainly Adult, 45 (IQR: 31–60) | SSI | Hospital-acquired | Hospital | 33 | 5 | CDD | Wound | 7 |
| [64] | Asante | 2019 | May-Sep 2015 | Ghana | Western | NA | Various | NA | Hospital | 91 | 43 | mecA | Blood, Urine | 5 |
| [65] | Wireko | 2021 | January 2019 - June 2020 | Ghana | Western | Adult, Older Adult, 18–87 | Filarial lymphedema | NA | Other | 31 | 21 | mecA | Wound, | 5 |
| [66] | Wolters | 2020 | January-November 2016 | Ghana | Western | Mainly Adult  ,≥15 | Wound | Outpatient | Hospital | 28 | 8 | mecA | Wound | 7 |
| [67] | Egyir | 2021 | June and November 2018. | Ghana | Western | All Ages | SSI | Hospital-acquired | Hospital | 13 | 4 | mecA | Wound, aspirate samples | 7 |
| [68] | Onuzo | 2022 | April to July 2017. | Ghana | Western | Adult, 28.3 ± 4.4 | SSI | Hospital-acquired | Hospital | 26 | 6 | ODD | Wound | 7 |
| [69] | Sanders | 2022 | NA | Ghana | Western | NA | SSI | Hospital-acquired | Hospital | 25 | 10 | mecA | Wound swabs and aspirates. | 6 |
| [70] | Maïga | 2017 | 2007 to 2009. | Mali | Western | NA | Various | NA | Hospital | 297 | 195 | CDD | Blood, Pus, Urine | 5 |
| [71] | Dicko | 2023 | January 2014 and December 2020 | Mali | Western | All Ages, 3–98 | Various | Outpatients and Inpatients | Hospital | 735 | 335 | CDD | Urine, Pus, Blood, vaginal secretions, pleurisy, catheters, Sputum, ascites, prostatic fluid. | 8 |
| [72] | Kalambry | 2024 | October 2021 to December 2022 | Mali | Western | Mainly Adult, Median = 37.5 | Infections associated with pleurisy | Inpatients | Hospital | 36 | 26 | CDD | Pleural effusion samples | 7 |
| [73] | Adetayo | 2014 | NA | Nigeria | Western | NA | Various | NA | Hospital | 23 | 7 | ODD | Urine, Wound swab, ear swab, and nasal swab | 5 |
| [74] | Alli | 2015 | March to June 2013 | Nigeria | Western | Mainly Adult, Mean = 27.14 | Various | Both  (SCCmec typing) | Hospital | 156 | 66 | mecA | Clinical isolates from various specimens | 7 |
| [75] | Yusuf | 2015 | September 2013 to November 2014 | Nigeria | Western | NA | Various | NA | Hospital | 522 | 223 | RLAT | Pus, Urine, Blood, Wound | 6 |
| [76] | Nsofor | 2016 | NA | Nigeria | Western | NA | Various | Outpatients | Hospital | 104 | 40 | ODD | Urine, Pus, vaginal, Wound, throat, Blood, stool | 6 |
| [77] | Osinupebi | 2018 | NA | Nigeria | Western | All Ages, 0–79 | Various | NA | Hospital | 161 | 66 | CDD | Various | 5 |
| [78] | Chukwueze | 2022 | NA | Nigeria | Western | Adolescents and Adults, 15–65 | Wound | Inpatients | Hospital | 188 | 86 | CDD | Wound | 7 |
| [79] | Udeani | 2016 | NA | Nigeria | Western | All Ages, 1–86 | Wound | NA | Hospital | 52 | 21 | CDD | Wound | 5 |
| [80] | Abdullahi | 2018 | April 2014 – August 2015 | Nigeria | Western | All Ages, 0–≥25 | Various | Outpatients and Inpatients | Hospital | 360 | 97 | CDD | Blood, Urine | 8 |
| [81] | AbdulAziz | 2022 | September 2017 – November 2018 | Nigeria | Western | Adult,  ≥18 | Wound | NA | Hospital | 73 | 63 | CDD | Wound | 5 |
| [82] | Olowo-Okere | 2017 | June 1, 2016 – August 30, 2016 | Nigeria | Western | All Ages ,0–≥37 | Wound | NA | Hospital | 20 | 5 | ODD | Wound | 5 |
| [83] | Mofolorunsho | 2022 | August 2017 – February 2018 | Nigeria | Western | All Ages, <16–>65 | Wound | NA | Hospital | 124 | 28 | ORSAB | Wound | 5 |
| [84] | Beshiru | 2023 | November 2022 – May 2023 | Nigeria | Western | NA | Wound | Outpatients and Inpatients | Hospital | 87 | 34 | mecA | Wound | 6 |
| [85] | Medugu | 2021 | June 2017 – May 2018 | Nigeria | Western | All Ages, <5–60 | Various | Outpatients and Inpatients | Hospital | 360 | 176 | CDD | Wound, Urine, Blood cultures, aspirates, and cervical swabs. | 8 |
| [86] | Ibadin | 2017 | NA | Nigeria | Western | NA | Various | Outpatients and Inpatients | Hospital | 50 | 19 | mecA | Wound, Blood, Urine | 6 |
| [87] | Ganau | 2019 | February – October 2015 | Nigeria | Western | NA | Various | Outpatients and Inpatients | Hospital | 234 | 101 | CDD | Wound | 6 |
| [88] | Olowe | 2013 | June – December 2010 | Nigeria | Western | NA | Various | NA | Hospital | 208 | 40 | mecA | Blood, Urine, Wound | 5 |
| [89] | Idris | 2019 | NA | Nigeria | Western | All Ages, 1–60 | Various | NA | Hospital | 252 | 65 | CDD | Blood, Wound, ear, CSF, throat, semen. | 5 |
| [90] | Oche | 2021 | September – December 2017 | Nigeria | Western | Pediatric, Adult, 1–≥40 | Various | Inpatients | Hospital | 26 | 17 | CDD | Wound, nasal, and Urine | 7 |
| [91] | Umar | 2023 | NA | Nigeria | Western | Pediatric, Adult ,1–50 | Various | Both (SCCmec typing) | Hospital | 90 | 36 | mecA | Wound, nasal, Urine, Pus, urethral, HVS, ear swabs, catheter tips | 7 |
| [92] | Okoye | 2022 | October 2017 – August 2018 | Nigeria | Western | NA | Various | NA | Hospital | 83 | 23 | RLAT | Urine, Wound, HVS, urethral swabs | 5 |
| [93] | Akanbi | 2013 | April 2010 – June 2011 | Nigeria | Western | NA | Various | Outpatients and Inpatients | Hospital | 214 | 28 | ODD | Wound, skin, Urine, Blood, vaginal, CSF, ear | 6 |
| [94] | Bawonda | 2024 | NA | Nigeria | Western | NA | Various | Inpatients | Hospital | 70 | 30 | CDD | Pus, throat, Wound, Urine, Sputum. | 6 |
| [95] | Oba | 2023 | NA | Nigeria | Western | Adult, 21–50 | SSI | Hospital-acquired | Hospital | 20 | 8 | CDD | Wound | 7 |
| [96] | Ejikeugwu | 2020 | NA | Nigeria | Western | NA | UTI | Community (Outpatients) | Hospital | 39 | 14 | CDD | Urine | 6 |
| [97] | Udobi | 2013 | NA | Nigeria | Western | NA | Wound | Inpatients | Hospital | 44 | 33 | ORSAB | Wound | 6 |
| [98] | Odogwu | 2019 | NA | Nigeria | Western | NA | Various | Outpatients and Inpatients | Hospital | 55 | 30 | mecA | Urine, HVS, ear, Wound swabs | 6 |
| [99] | Bunza | 2019 | June – September 2018 | Nigeria | Western | children, adolescents, and adults, 1–50 | Various | Inpatients | Hospital | 100 | 66 | CDD | Urine, Wound, Pus, HVS, nasal, ear swabs | 7 |
| [100] | Yahaya | 2022 | June – August 2021 | Nigeria | Western | NA | Various | Outpatients and Inpatients | Hospital | 31 | 10 | CDD | Wound, Urine, Blood, Sputum | 6 |
| [101] | Obasuyi | 2015 | July – September 2007 | Nigeria | Western | NA | Various | NA | Hospital | 75 | 43 | Oxacillin E-test | Ear, Urine, cervical swabs, Blood, Wound | 5 |
| [102] | Schaumburg | 2022 | July 2019 to 30 November 2020 | Sierra Leone | Western | All Ages ,0–88 | Chronic Wound infections | NA | Hospital | 31 | 17 | mecA | Wound | 5 |
| [103] | Dossim | 2024 | January 1, 2018, to December 31, 2019, | Togo | Western | Mainly Pediatrics | Various | Inpatients | Hospital | 123 |  | CDD | Pus, Urine, Blood | 7 |
| [104] | Darboe | 2019 | 2005 - December 2015 | Gambia | Western | Pediatric,1–21 | SSTIs and BSI | Community-acquired (72 Hour cutoff) | Hospital | 293 | 7 | CDD | Blood and Pus, Wound | 7 |
| [105] | Gouleu | 2024 | 2009 – 2019 | Gabon | Central | All Ages, 0–76 | SSTI | Outpatients and Inpatients | Hospital | 499 | 36 | CDD | Swabs, Pus | 8 |
| [106] | Alabi | 2013 | 2009–2012 | Gabon | Central | All Ages, 0–89 | Various | NA | Hospital | 326 | 19 | CDD | Blood, Wound, Urine, and other body fluids | 6 |
| [107] | Schaumburg | 2011 | 2008–2010 | Gabon | Central | Mainly Adult, Mean = 40.3 | Various | NA | Hospital | 44 | 6 | mecA | Wound, Blood, abscesses | 5 |
| [108] | Dikoumba | 2021 | January 2016 – March 2018 | Gabon | Central | All Ages, 0–79 | Various | Outpatients and Inpatients | Hospital | 35 | 6 | CDD | Blood, Urine, Pus | 7 |
| [109] | Okuda | 2016 | 2012 – 2013 | Gabon | Central | All Ages | SSTI | Outpatients | Hospital | 24 | 3 | mecA | Pus | 7 |
| [110] | Vandendriessche | 2017 | 2009 – 2013 | DR Congo | Central | All Ages ,0–90 | BSI | Inpatients | Hospital | 108 | 27 | mecA | Blood | 7 |
| [111] | Iyamba | 2014 | January – October 2013 | DR Congo | Central | NA | SSI | Inpatients | Hospital | 74 | 47 | ODD | Wound | 6 |
| [112] | Mohamadou | 2022 | April 2019 – Dec 2020 | Cameroon | Central | All Ages, 0–85 | Various | Both (SCCmec typing) | Hospital | 380 | 104 | mecA | Pus, Urine, Blood | 8 |
| [113] | Bissong | 2016 | March – June 2016 | Cameroon | Central | All Ages, ≤20–>60 | Various | Inpatients | Hospital | 33 | 15 | ODD | Pus, ear, genital swabs | 7 |
| [114] | Foloum | 2021 | 2017 – 2019 | Cameroon | Central | All Ages, 0–84 | Various | NA | Hospital | 90 | 59 | CDD | Blood, Urine, uro-genital, Wound | 5 |
| [115] | Manhafo | 2021 | Jan – May 2021 | Cameroon | Central | Mainly Adult, Median31.5 (IQR: 19–40) | SSTIs | Community | Hospital | 22 | 9 | CDD | Pus | 7 |
| [116] | Massongo | 2021 | 2010 and 2017 | Cameroon | Central | Mainly Adult,  Mean=30.2 ± 21.0 | Various | NA | Hospital | 824 | 282 | CDD | Blood, Sputum, Urine, and others | 6 |
| [117] | Kengne | 2024 | 2021 to March 2023 | Cameroon | Central | Mainly Adult, Mean=49.5 ± 14.6 | Various | Outpatients and Inpatients | Hospital | 81 | 62 | CDD | Fecal samples | 7 |
| [118] | Fonkoue | 2024 | 2016 to August 2023 | Cameroon | Central | All Ages, 5–85 | Fracture-related infections | Outpatients and Inpatients | Hospital | 69 | 43 | ODD | Deep tissue samples | 7 |
| [119] | Ndedy | 2023 | May 12 to July 28, 2020 | Cameroon | Central | All Ages, ≤20–>60 | Various | NA | Hospital | 70 | 43 | mecA | Wound, nasopharynx, Urine | 5 |
| [120] | Kengne (a) | 2019 | January 2014 to November 2016. | Cameroon | Central | NA | Various | Outpatients and Inpatients | Hospital | 250 | 201 | CDD | Blood, Pus, Urine, ear, throat, urethral, vaginal swabs, semen. | 6 |
| [121] | Gaké | 2022 | NA | Cameroon | Central | All Ages, 06–67 | Various | NA | Hospital | 200 | 84 | CDD | Stool, Urine, Pus | 5 |
| [122] | Abdoul-Latif | 2022 | 2019–2021 | Djibouti | Eastern | NA | DFI | Both | Hospital | 110 | 49 | ODD | Pus, Blood, Sputum | 6 |
| [123] | Garoy | 2019 | 2016 | Eritrea | Eastern | All Ages, 3–67 | SSTI | Hospital-acquired (72 Hour cutoff) | Hospital | 82 | 59 | ODD | Pus, Blood | 7 |
| [124] | Garoy (a) | 2021 | February–May 2017 | Eritrea | Eastern | All Ages, 2–82 | SSI | Hospital-acquired | Hospital | 20 | 14 | ODD | Wound | 7 |
| [125] | Kahsay | 2014 | Dec 2011–Mar 2012 | Ethiopia | Eastern | Mainly Adult, Mean=35 ± 14.4 | SSI | Hospital-acquired | Hospital | 73 | 36 | ODD | Wound | 7 |
| [126] | Godebo | 2013 | June–Dec 2011 | Ethiopia | Eastern | NA | Wound | Outpatients and Inpatients | Hospital | 73 | 21 | CDD | Wound | 6 |
| [127] | Mitiku | 2021 | July–October 2020 | Ethiopia | Eastern | All Ages, 18–89 | UTI | Outpatients | Hospital | 54 | 23 | CDD | Urine | 7 |
| [128] | Tsige | 2020 | Feb–May 2016 | Ethiopia | Eastern | All Ages, 5–81 | Wound | NA | Hospital | 92 | 26 | CDD | Wound | 5 |
| [129] | Dilnessa | 2016 | Sep 2013–Apr 2014 | Ethiopia | Eastern | All Ages ,0–89 | Various | NA | Hospital | 194 | 34 | CDD | Pus, nasal, Blood | 5 |
| [130] | Kejela | 2022 | Nov 2019–Apr 2020 | Ethiopia | Eastern | NA | Wound | Inpatients | Hospital | 126 | 72 | mecA | Wound | 6 |
| [131] | Worku | 2023 | July 2020–Aug 2021 | Ethiopia | Eastern | All Ages, 0–85 | SSI | Hospital-acquired | Hospital | 163 | 40 | CDD | Wound | 5 |
| [132] | Tefera | 2021 | Feb–Apr 2020 | Ethiopia | Eastern | All Ages, 1–84 | Wound | Inpatients | Hospital | 71 | 32 | CDD | Wound | 7 |
| [133] | Tadesse | 2018 | Dec 2013–June 2014 | Ethiopia | Eastern | All Ages, <11–≥41 | SSI and ear infections | Outpatients and Inpatients | Hospital | 79 | 54 | CDD | Wound, ear swabs | 7 |
| [134] | Moges | 2023 | 2017–2018 | Ethiopia | Eastern | All Ages, <5–>60 | Various | NA | Hospital | 139 | 38 | CDD | Blood, Wound, Urine | 5 |
| [135] | Asefa | 2014 | 2008–2014 | Ethiopia | Eastern | All Ages | Various | NA | Hospital | 238 | 106 | Cefoxitin E-test | Pus, Blood, ear discharge | 5 |
| [136] | Abebe | 2024 | Nov 2020–Apr 2021 | Ethiopia | Eastern | All Ages, 1–79 | Wound | NA | Hospital | 109 | 44 | CDD | Wound | 5 |
| [137] | Mama | 2019 | April–June 2017 | Ethiopia | Eastern | All Ages, ≤15–≥60 | Wound | NA | Hospital | 79 | 65 | CDD | Wound | 5 |
| [138] | Wangai | 2019 | 2014–2016 | Kenya | Eastern | Mainly Adult,  Median = 48 | SSTI | NA | Hospital | 187 | 100 | CDD | Skin, Blood, Urine | 5 |
| [139] | Akoru | 2016 | 2010–2011 | Kenya | Eastern | NA | Various | NA | Hospital | 107 | 39 | RLAT | Pus, tracheal aspirate | 5 |
| [140] | Gitau | 2018 | 2014–2016 | Kenya | Eastern | NA | Various | NA | Hospital | 944 | 262 | CDD | Pus, Blood, tracheal aspirates | 6 |
| [141] | Iliya | 2020 | 2017–2018 | Kenya | Eastern | All Ages, 1–80 | Various | Outpatients and Inpatients | Hospital | 54 | 22 | CDD | Pus, Tracheal Aspirate, Urine, Blood, Ascitic Fluid, CSF, Peritoneal, Synovial Fluid | 7 |
| [142] | Okello | 2021 | 2014–2018 | Kenya | Eastern | Mainly Adult, Mean = 29.66 | Various | Inpatients | Hospital | 659 | 4 | ODD | Pus, CSF, Blood | 8 |
| [143] | Omuse | 2014 | 2011–2013 | Kenya | Eastern | NA | Various | NA | Hospital | 731 | 24 | CDD | Pus, Blood, Urine | 6 |
| [144] | Maina | 2013 | 2005–2007 | Kenya | Eastern | Adult, ≥18 | SSTI | Outpatients and Inpatients | Hospital | 82 | 69 | CDD | Skin, soft tissue | 7 |
| [145] | Musicha | 2017 | 1998–2016 | Malawi | Eastern | Pediatric, Adult, <5–45 | BSI | Community-acquired | Hospital | 1923 | 107 | CDD | Blood cultures | 8 |
| [146] | Choonara | 2022 | June – December 2017 | Malawi | Eastern | All Ages, 18–89 | Various | NA | Hospital | 86 | 9 | mecA | Pus, Blood, Urine | 5 |
| [147] | Nuckchady | 2020 | 2015–2016 | Mauritius | Eastern | Adult, ≥18 | Various | Inpatients | Hospital | 15 | 8 | CDD | Blood, Urine, Pus | 7 |
| [148] | Vubil | 2017 | 2001–2009 | Mozambique | Eastern | Pediatric, <5 | BSI | Outpatients | Hospital | 84 | 7 | ODD | Blood | 7 |
| [149] | Kenga | 2021 | 2016–2019 | Mozambique | Eastern | Pediatric | BSI | Community-acquired | Hospital | 35 | 25 | CDD | Blood | 7 |
| [150] | Meeren | 2014 | 2010–2011 | Mozambique | Eastern | All Ages, 1–76 | Wound and SSTIs | Outpatients and Inpatients | Hospital | 99 | 9 | mecA | Pus, Wound | 7 |
| [151] | Garrine | 2023 | 2001–2019 | Mozambique | Eastern | Pediatric, <5 | BSI | NA | Hospital | 336 | 16 | CDD | Blood | 6 |
| [152] | Ishimwe | 2018 | 2015 | Rwanda | Eastern | Pediatric, 0.2–15 | BSI | NA | Hospital | 22 | 13 | ODD | Blood | 7 |
| [153] | Masaisa | 2018 | 2013–2014 | Rwanda | Eastern | All Ages, 0.7–73 | Various | Both (Outpatients and Inpatients, SCCmec typing) | Hospital | 138 | 39 | mecA | Pus, Blood | 7 |
| [154] | Ntirenganya | 2015 | 2013 | Rwanda | Eastern | All Ages, 15–89 | Various | NA | Hospital | 22 | 18 | ODD | Various | 7 |
| [155] | Sutherland | 2019 | 2017 | Rwanda | Eastern | Adult, ≥15 | Various | Inpatients | Hospital | 22 | 7 | CDD | Blood, Urine, Wound | 7 |
| [156] | Noel | 2017 | Apr–Jul 2014 | Rwanda | Eastern | All Ages, <10–<50 | Various | NA | Hospital | 30 | 19 | ODD | Blood, Pus | 5 |
| [157] | Kumburu | 2018 | August 2013 to August 2015 | Tanzania | Eastern | NA | Various | NA | Hospital | 30 | 10 | CDD | Wound, Blood, Sputum | 5 |
| [158] | Manyahi | 2014 | September 2011 to February 2012 | Tanzania | Eastern | All Ages, 18–80 | SSI |  | Hospital | 18 | 8 | CDD | Wound | 5 |
| [159] | Onken | 2015 | March 2012 to April 2013 | Tanzania | Eastern | All Ages | BSI | NA | Hospital | 9 | 0 | CDD | Blood | 5 |
| [160] | Ernest | 2024 | March to April 2023 | Tanzania | Eastern | Pediatric, <5 | UTI | NA | Hospital | 26 | 6 | CDD | Urine samples | 5 |
| [161] | Moremi | 2016 | June 2013 to May 2015 | Tanzania | Eastern | All Ages | Various | NA | Hospital | 100 | 27 | CDD | Blood | 5 |
| [162] | Kamori | 2024 | April to June 2022 | Tanzania | Eastern | Adult, ≥18 | pneumonia | Inpatients and Outpatients | Hospital | 22 | 11 | CDD | Sputum | 7 |
| [163] | Mwailunga | 2023 | April to June 2023 | Tanzania | Eastern | Mainly Adolescent ,Median = 17 | systemic infections | NA | Hospital | 12 | 9 | CDD | Blood, CSF, other clinical samples | 5 |
| [164] | Mnyambwa | 2021 | October 2018 to September 2019 | Tanzania | Eastern | Mainly Adult, Median = 28 | Various | Outpatients and Inpatients | Hospital | 33 | 22 | CDD | Ear Pus, Urine, Wound Pus, stool, and Blood | 7 |
| [165] | Geofrey | 2024 | NA | Tanzania | Eastern | Mainly Adult, Median = 29 (IQR 20–45) | Various | Outpatients and Inpatients | Hospital | 103 | 50 | mecA | Pus, Wound, Blood, Urine, stool, peritoneal fluid, and Sputum | 7 |
| [166] | Moremi | 2014 | November 2011 to February 2012 | Tanzania | Eastern | NA | CLLUs | NA | Hospital | 18 | 8 | CDD | Wound |  |
| [167] | Mushi | 2015 | October 2013 to March 2014 | Tanzania | Eastern | All Ages | CSOM | NA | Hospital | 34 | 14 | CDD | Ear swabs |  |
| [168] | Johnson | 2021 | November 2019 - February 2020 | Uganda | Eastern | Adult (Pregnant) | UTI | NA | Hospital | 33 | 11 | ODD | Urine | 5 |
| [169] | Pius | 2023 | Not specified | Uganda | Eastern | All Ages, 0–84 | SSTI | Outpatients and Inpatients | Hospital | 78 | 42 | ODD | Wound | 7 |
| [170] | George | 2018 | June to October 2015 | Uganda | Eastern | All Ages ,2–80 | SSI | Hospital-acquired | Hospital | 41 | 27 | ODD | Pus | 7 |
| [171] | Wekesa | 2020 | November 2017 - April 2018 | Uganda | Eastern | Mainly Adult, Median=25 | SSI | Hospital-acquired | Hospital | 34 | 31 | mecA | Wound | 7 |
| [172] | Andrew | 2016 | June and November 2014 | Uganda | Eastern | All Ages | SSI | Hospital-acquired | Hospital | 331 | 179 | CDD | Pus swabs | 8 |
| [173] | Seni | 2013 | September 2011 to April 2012 | Uganda | Eastern | Mainly Adult, Mean=25±13.14 | SSI | Hospital-acquired | Hospital | 64 | 24 | mecA | Wound | 7 |
| [174] | Iramiot | 2014 | June 2012 to June 2013 | Uganda | Eastern | NA | Various | NA | Hospital | 300 | 114 | mecA | Various | 6 |
| [175] | Mwansa | 2022 | January 2019 - December 2021 | Zambia | Southern | All Ages, 0–80 | Various | Outpatients and Inpatients | Hospital | 38 | 9 | CDD | Blood, Urine, Sputum | 7 |
| [176] | Shawa | 2024 | 2015-2020 | Zambia | Southern | All Ages | Various | NA | Hospital | 2181 | 973 | CDD | Blood, Urine, Wound swabs | 6 |
| [177] | Samutela | 2015 | June 2009 and December 2012 | Zambia | Southern | NA | Various | NA | Hospital | 95 | 41 | CDD | Blood, Urine, Wound | 5 |
| [178] | Roth | 2021 | July 2015 - April 2017 | Zambia | Southern | All Ages | Various | Outpatients and Inpatients | Hospital | 109 | 40 | CDD | Blood, Urine, Nasal swabs | 7 |
| [179] | Simango | 2019 | NA | Zimbabwe | Southern | All Ages | Various | Outpatients and Inpatients | Hospital | 381 | 45 | CDD | Blood, Urine, Wound | 8 |
| [180] | Mauchaza | 2016 | June 2013 - May 2014 | Zimbabwe | Southern | NA | Various | Outpatients and Inpatients | Hospital | 407 | 30 | CDD | Blood, Pus, Sputum, others | 7 |
| [181] | Iileka | 2016 | 2012-2014 | Namibia | Southern | All Ages | Various | NA | Hospital | 600 | 81 | ODD | Blood, Sputum, Wound | 6 |
| [182] | Naidoo | 2013 | January 2007 - December 2011 | South Africa | Southern | All Ages,0–87 | BSI | Outpatients and Inpatients | Hospital | 365 | 95 | ODD | Blood | 8 |
| [183] | Oosthuysen | 2013 | 2009-2010 | South Africa | Southern | All Ages, 0–87 | Various | NA | Hospital | 367 | 56 | mecA | Skin, soft tissue, | 6 |
| [184] | Abdulgader | 2020 | 2009-2011 & 2015-2017 | South Africa | Southern | NA | Various | NA | Hospital | 212 | 93 | CDD | Blood, Pus swabs | 5 |
| [185] | Perovic (a) | 2017 | January 2013 - January 2016 | South Africa | Southern | All Ages | BSI | Both (SCCmec typing) | Hospital | 1914 | 557 | mecA | Blood | 8 |
| [186] | Shuping | 2017 | January 2014 - December 2014 | South Africa | Southern | All Ages ,<1 month–≥65 | BSI | Hospital-acquired (48-hour criterion) | Hospital | 772 | 231 | ODD | Blood | 7 |
| [187] | Perovic | 2015 | June 2010 - July 2012 | South Africa | Southern | All Ages | BSI | Both (SCCmec typing) | Hospital | 2709 | 1231 | mecA | Blood | 8 |
| [188] | Singh-Moodley | 2021 | 2016–2017. | South Africa | Southern | NA | BSI | Hospital-acquired (SCCmec typing) | Hospital | 1543 | 374 | mecA | Blood | 7 |
| [189] | Fortuin-de Smidt | 2015 | September 2012 – September 2013 | South Africa | Southern | All Ages, 1–50 | BSI | Both (3 days) | Hospital | 442 | 86 | ODD | Blood | 8 |
| [190] | Strasheim | 2021 | 2013–2017 | South Africa | Southern | All Ages | BSI | Hospital-acquired | Hospital | 1239 | 329 | mecA | Blood | 8 |
| [191] | Mahomed | 2018 | Oct 2013–May 2014 | South Africa | Southern | All Ages, 1–40 | cystic fibrosis | NA | Hospital | 33 | 17 | mecA | Sputum | 5 |
| UTI: Urinary Tract Infection; DFI: Diabetic Foot Infection; CLLUs: Chronic Lower Limb Ulcers; CSOM: Chronic Suppurative Otitis Media; NA: Not Available; ODD: Oxacillin Disk Diffusion; CDD: Cefoxitin Disk Diffusion; CSF: Cerebrospinal Fluid; HVS: High Vaginal Swab; BSI: Bloodstream Infection; SSI: Surgical Site Infection; SSTI: Skin and Soft Tissue Infection. | | | | | | | | | | | | | | |

**References**

1. Djahmi N, Messad N, Nedjai S, Moussaoui A, Mazouz D, Richard JL, et al. Molecular epidemiology of Staphylococcus aureus strains isolated from Inpatients with infected diabetic foot ulcers in an Algerian University Hospital. Clin Microbiol Infect. 2013;19 (9):398–404.

2. Aouati H, Hadjadj L, Aouati F, Agabou A, Khedher M Ben, Bousseboua H, et al. Emergence of methicillin-resistant staphylococcus aureus ST239/241 SCCmec-III mercury in Eastern Algeria. Pathogens. 2021;10 (11):1503.

3. Tchakal-Mesbahi A, Abdouni MA, Metref M. Prevalence Of Multidrug-Resistant Bacteria Isolated From Burn Wound In Algeria. Ann Burns Fire Disasters. 2021;34 (2):150–6.

4. Rahima T, Nafissa B, Abdelghani D. Prevalence of methicillin-resistant Staphylococcus aureus and/or intermediate susceptibility to vancomycin isolated from private laboratories in Annaba “Algeria.” J Chem Pharm Res. 2015;7 (5):780–6.

5. Achek R, Hotzel H, Cantekin Z, Nabi I, Hamdi TM, Neubauer H, et al. Emerging of antimicrobial resistance in staphylococci isolated from clinical and food samples in Algeria. BMC Res Notes. 2018;11 (1):1–7.

6. Aidaoui S, Hecini-Hannachi A. Epidemiology and antibiotic susceptibility of multidrug-resistant Staphylococcus aureus in nosocomial and community acquired infections in southeastern Algeria. South Asian J Exp Biol. 2022;12 (6):811–9.

7. Rahmani A, Meradi L, Malawi K, Khanfouf F. Phenotypic characterization of antimicrobial drug resistance of Staphylococcus aureus and S. epidermidis strains isolated from various community infections in Oum El Bouaghi city, Algeria. Biodiversitas J Biol Divers. 2021;22 (5):2665–71.

8. Aiza A, Kaidi R, Ahmed M, Khiati B. Bacteriological Profile and Antibiotic Resistance Patterns of Pus/Wound Samples in Humans with Infected Wound in North Central Algeria. J Pure Appl Microbiol. 2023;17 (3):1628–40.

9. Bouharkat B, Tir Touil A, Mullié C, Chelli N, Meddah B. Bacterial ecology and antibiotic resistance mechanisms of isolated resistant strains from diabetic foot infections in the north west of Algeria. J Diabetes Metab Disord. 2020;19 (2):1261–71.

10. Hecini-Hannachi A, Bentchouala C, Lezzar A, Laouar H, Benlabed K, Smati F. Multidrug-resistant bacteria isolated from patients hospitalized in Intensive Care Unit in University Hospital of Constantine, Algeria (2011 - 2015). African J Microbiol Res. 2016;10 (33):1328–36.

11. Benyagoub E. Methicillin, β-lactams, and Clindamycin Resistance Profiles of Staphylococcus aureus Strains Isolated from Patients with UTI in Bechar Province (Algeria). Anti-Infective Agents. 2024;22 (1):54–65.

12. Warag S, Foster H, Daw M. Antimicrobial susceptibility patterns of Methicillin-resistant staphylococcus aureus isolates collected from healthcare and community facilities in Libya show a high level of resistance to fusidic acid. J Infect Dis Ther. 2014;2 (6):2–6.

13. Ahmad KM, Alamen AA, Atiya FA, Elzen AA. Prevalence of Methicillin-resistant Staphylococcus Aureus (MRSA) Among Staphylococcus Aureus Collection at Sebha Medical Center. J Adv Lab Res Biol. 2018;9 (1):01–8.

14. Aetrugh S, Aboshkiwa M, Husien W, Erhuma M, Corrente M, Grandolfo E, et al. Antimicrobial resistance profile and molecular characterization of methicillin-resistant staphylococcus isolates in Tripoli Central Hospital, Libya. Libyan Int Med Univ J. 2017;2 (1):74–83.

15. Eldukali WA, Boshaalla MA. Diabetic Foot Infection Characteristics and Antibiotics Susceptibility Patterns in a Regional Hospital in Libya. Ibnosina J Med Biomed Sci. 2022;14 (3):094–100.

16. Elhassan MM, Ozbak HA, Hemeg HA, Elmekki MA, Ahmed LM. Absence of the mecA Gene in Methicillin Resistant Staphylococcus aureus Isolated from Different Clinical Specimens in Shendi City, Sudan. Biomed Res Int. 2015;2015 (1):895860–5.

17. Abdalla AE, Kabashi AB, Elobaid ME, Haj Hamed NM, Modawyi WA, Mohammed Alameen AA, et al. Methicillin and inducible Clindamycin-Resistant Staphylococcus aureus Isolated from Postoperative Wound samples. J Pure Appl Microbiol. 2019;13 (3):1605–9.

18. Hamdan EM, Hassan AA, Amin SA, Ahmed HA, Ataelmanan AE, Elmakki MA, et al. Increasing Prevalence of Methicillin-resistant Staphylococcus aureus among Hospital and Community Acquired Infections in Khartoum State, Sudan. Infect Disord - Drug Targets. 2022;23:1–1.

19. Elimam MAE, Rehan S, Elmekki MA, Elhassan MM. Emergence of vancomycin resistant and methcillin resistant staphylococus aureus in patients with different clinical manifestations in Khartoum state, Sudan. J Am Sci. 2014;10 (6):106–10.

20. Babiker W, Ahmed A, Babiker T, Ibrahim EM, Almugadam BS. Prevalence and Causes of Neonatal Sepsis in Soba University Hospital, Sudan. Med Microbiol Rep. 2018;1 (2):11–3.

21. Moglad EH, Altayb HN. Antibiogram, prevalence of methicillin-resistant and multi-drug resistant Staphylococcus spp. in different clinical samples. Saudi J Biol Sci. 2022;29 (12):103432–7.

22. Abdelaziz M, Hamadalnil Y, Hashim O, Bashir T, Mahjoub ES. Microbiological profile of neonatal sepsis at a maternity hospital in Omdurman, Sudan. Sudan J Med Sci. 2019;14 (1):45–51.

23. Osman NAM, Elraya IE, Mohamed YM, Eldirdery MM, Elzaki SG, Ahmed AE, et al. Frequency of Methicillin Resistance Among Staphylococcus Aureus Clinical Isolates in Khartoum State, Sudan. Sudan J Med Sci. 2018;13 (4):240–50.

24. Ibrahim OM, Azoz ME. The Prevalence of Methicillin resistant Staphylococcus aureus among Surgical Patients at Kosti Teaching Hospital , Kosti – Sudan. Eur Acad Res. 2017;5 (6):2566–79.

25. Alhady SAA, Ali MA. The Prevalence of Inducible Clindamycin Resistance Staphylococcus aureus among Various Clinical Specimens in Khartoum state Sudan. Int J Sci Res Sci Eng Technol. 2021;8 (5):13–21.

26. Aniba R, Barguigua A, Dihmane A, Momen G, Nayme K, Timinouni M. Prevalence and antibacterial resistance patterns of uropathogenic staphylococci in Casablanca, Morocco. J Infect Dev Ctries. 2023;17 (10):1436–45.

27. Serray B, Zriouil SB, Sobh M, Soraa N, Saile R, Hammoumi A, et al. View of Molecular and phenotypic characteristics of methicillin-resistant Staphylococcus aureus isolated from Pediatric Hospital in Morocco. Moroccan J Public Heal. 2022;4 (1):59–67.

28. Mourabit N, Arakrak A, Bakkali M, Laglaoui A. Prevalence of MRSA and Panton-Valentine Leukocidin-Positive strains identified at the trauma service of Tangier Morocco. Am J Res Commun. 2014;2 (9):163–74.

29. Elbargisy RM. Distribution of Leukocidins, Exfoliative Toxins, and Selected Resistance Genes Among Methicillin-resistant and Methicillin-sensitive Staphylococcus aureus Clinical Strains in Egypt. Open Microbiol J. 2022;16 (1):1–9.

30. Sonbol FI, Abdelaziz AA, El-banna TE, Farag O. Detection and Characterization of Staphylococcus aureus and Methicillin-resistant S. aureus (MRSA) in Ear Infections in Tanta, Egypt. J Adv Med Pharm Res. 2022;3 (2):36–44.

31. AbdEl-Mongy M, Awad ET, Mosaed F. Vancomycin resistance among methicillin resistant Staphylococcus aureus isolates from neonatal sepsis attending intensive care unit in Shibin El-Kom Teaching Hospital, Egypt. J Pure Appl Microbiol. 2018;12 (3):1093–100.

32. Hashem RA, Yassin AS, Zedan HH, Amin MA. Fluoroquinolone resistant mechanisms in methicillin-resistant Staphylococcus aureus clinical isolates in Cairo, Egypt. J Infect Dev Ctries. 2013;7 (11):796–803.

33. Alfeky AAE, Tawfick MM, Ashour MS, El-Moghazy ANA. High Prevalence of Multi-drug Resistant Methicillin-Resistant Staphylococcus aureus in Tertiary Egyptian Hospitals. J Infect Dev Ctries. 2022;16 (5):795–806.

34. Omar NY, Ali HAS, Harfoush RAH, El Khayat EH. Molecular Typing of Methicillin Resistant Staphylococcus aureus Clinical Isolates on the Basis of Protein A and Coagulase Gene Polymorphisms. Int J Microbiol. 2014;2014 (1):650328–39.

35. El-baz R, Rizk DE, Barwa R, Hassan R. Virulence characteristics and molecular relatedness of methicillin resistant Staphylococcus aureus harboring different staphylococcal cassette chromosome mec. Microb Pathog. 2017;113:385–95.

36. Kadry A, Shaker G, El-Ganiny A, Youssef C. Phenotypic and Genotypic detection of local MRSA isolates. Zagazig J Pharm Sci. 2016;25 (1):39–46.

37. Sultan AM, Nabiel Y. Association of tsst-1 and pvl with mecA Genes among Clinical Staphylococcus aureus Isolates from a Tertiary Care hospital. J Pure Appl Microbiol. 2019;13 (2):855–64.

38. Barakat GI, Nabil YM. Correlation of mupirocin resistance with biofilm production in methicillin-resistant Staphylococcus aureus from surgical site infections in a tertiary centre, Egypt. J Glob Antimicrob Resist. 2016;4:16–20.

39. Shebl HR, Zaki WK, Saleh AN, Abdel Salam SA. Prevalence of MecC gene among methicillin resistant staphylococcus aureus isolated from patients in Ainshams University Hospital. J Pure Appl Microbiol. 2020;14 (4):2807–13.

40. El-Sweify MA, Raheel AS, Abu-Ata HN, El-Hadidy GS, Hessam WF. Identification of community-acquired methicillin-resistant Staphylococcus aureus (CA-MRSA) causing hospital-acquired infections in Suez Canal University Hospitals, Egypt by detection of its major virulence determinants. Microbes Infect Dis. 2021;2 (4):715–24.

41. Kishk RM, Mandour MF, Saleh RM. Staphylococcal Cassette Chromosome mec (SCCmec) Gene Typing in Detection of Methicillin-Resistant Staphylococcus aureus: Toward Precise Detection in Health Care Facility. Open J Med Microbiol. 2019;9 (3):127–37.

42. Mashaly M, El-Mashad N, El-deeb H. Detection of VanA type vancomycin resistance among MRSA isolates from an emergency hospital in Egypt. Comp Clin Path. 2019;28 (4):971–6.

43. Youssef CRB, Kadry AA, El-Ganiny AM. Investigating the relation between resistance pattern and type of Staphylococcal cassette chromosome mec (SCC mec) in methicillin-resistant Staphylococcus aureus. Iran J Microbiol. 2022;14 (1):56–66.

44. Shrief R, El Kholy RM, Rizk MA, Zaki ME. Prevalence of Tetracycline Resistant Genes in Staphylococcus aureusIsolates from Surgical Site Infections Egypt. Biosci Biotechnol Res Asia. 2019;16 (2):221–8.

45. Elkhyat AH, Makled AF, Albeltagy AM, Keshk TF, Dawoud AM. Prevalence of vanA Gene among Methicillin Resistant S. aureus Strains Isolated from Burn Wound Infections in Menoufia University Hospitals. Egypt J Med Microbiol. 2020;29 (3):97–104.

46. Taha AE, Badr MF, El-Morsy FE, Hammad E. Prevalence and antimicrobial susceptibility of methicillin-resistant staphylococcus aureus in an Egyptian University Hospital. J Pure Appl Microbiol. 2019;13 (4):2111–22.

47. Abdelraheem WM, Khairy RMM, Zaki AI, Zaki SH. Effect of ZnO nanoparticles on methicillin, vancomycin, linezolid resistance and biofilm formation in Staphylococcus aureus isolates. Ann Clin Microbiol Antimicrob. 2021;20 (1):1–11.

48. Zaki M, Galeb S, Eid AR, Ahmed D, Mabrouk A, Latif RA. Molecular characterization of Staphylococcus aureus isolated from hospital acquired sepsis in pediatrics, relation to antibiotics, resistance and virulence genes. Germs. 2020;10 (4):295–302.

49. El Gemezy E, Serry F, Kadry A. Antimicrobial susceptibility of Staphylococcus aureus clinical isolates and prevalence of MRSA in ICUs of Mansoura University Hospitals. Zagazig J Pharm Sci. 2016;25 (2):93–7.

50. Fahim NA. Prevalence and antimicrobial susceptibility profile of multidrug-resistant bacteria among intensive care units patients at Ain Shams University Hospitals in Egypt-a retrospective study. J Egypt Public Health Assoc. 2021;96 (1).

51. Ali AM, Sayed NM, Hassan RA. Study of vancomycin susceptibility pattern among Staphylococcus aureus isolated from superficial incisional surgical site infections. Microbes Infect Dis. 2022;3 (2):309–17.

52. Sleem AS, Ajlan SE, Zaher EM, Elmahdy EE. Phenotypic and genotypic detection of antimicrobial resistance and virulence factors among Staphylococcus aureus clinical isolates. Microbes Infect Dis. 2022;3 (4):910–9.

53. Ibrahim ESH, El-Baghdady K, Abd El-All SM, Warda MAA, Prince AM, Ibrahim M. Prevalence of multidrug resistance in the Egyptian methicillin-resistant Staphylococcus aureus isolates. African J Biol Sci. 2020;16 (1):43–52.

54. Hassan RH, Eldegla H, Elmorsy F, Eldars WM. Clinical and microbiological characteristics of healthcare-associated infections in a tertiary care pediatric hospital. Egypt Pediatr Assoc Gaz. 2017;65 (4):127–31.

55. Yehouenou CL, Kpangon AA, Affolabi D, Rodriguez-Villalobos H, Van Bambeke F, Dalleur O, et al. Antimicrobial resistance in hospitalized surgical patients: a silently emerging public health concern in Benin. Ann Clin Microbiol Antimicrob. 2020;19 (1):1–10.

56. Lai PS, Bebell LM, Meney C, Valeri L, White MC. Epidemiology of antibiotic-resistant Wound infections from six countries in Africa. BMJ Glob Heal. 2018;2 (4).

57. Ombelet S, Kpossou G, Kotchare C, Agbobli E, Sogbo F, Massou F, et al. Blood culture surveillance in a secondary care hospital in Benin: epidemiology of Bloodstream infection pathogens and antimicrobial resistance. BMC Infect Dis. 2022;22 (1):1–15.

58. Monteiro T, Wysocka M, Tellez E, Monteiro O, Spencer L, Veiga E, et al. A five-year retrospective study shows increasing rates of antimicrobial drug resistance in Cabo Verde for both Staphylococcus aureus and Escherichia coli. J Glob Antimicrob Resist. 2020;22:483–7.

59. Egyir B, Guardabassi L, Sørum M, Nielsen SS, Kolekang A, Frimpong E, et al. Molecular Epidemiology and Antimicrobial Susceptibility of Clinical Staphylococcus aureus from Healthcare Institutions in Ghana. PLoS One. 2014;9 (2):e89716.

60. Anafo RB, Atiase Y, Dayie NTKD, Kotey FCN, Tetteh-Quarcoo PB, Duodu S, et al. Methicillin-Resistant Staphylococcus aureus (MRSA) Infection of Diabetic Foot Ulcers at a Tertiary Care Hospital in Accra, Ghana. Pathogens. 2021;10 (8):937.

61. Karikari AB, Frimpong E, Owusu-Ofori A. Methicillin-resistant Staphylococcus aureus among patients in a teaching hospital in Ghana. Int J One Heal. 2017;3:46–9.

62. Opintan JA, Newman MJ. Prevalence of antimicrobial resistant pathogens from Blood cultures: Results from a laboratory based nationwide surveillance in Ghana. Antimicrob Resist Infect Control. 2017;6 (1):1–6.

63. Bediako-Bowan AAA, Kurtzhals JAL, Mølbak K, Labi AK, Owusu E, Newman MJ. High rates of multi-drug resistant gram-negative organisms associated with surgical site infections in a teaching hospital in Ghana. BMC Infect Dis. 2020;20 (1):1–9.

64. Asante J, Govinden U, Owusu-Ofori A, Bester LA, Essack SY. Molecular characterization of methicillin-resistant Staphylococcus aureus isolates from a hospital in Ghana. African J Clin Exp Microbiol. 2019;20 (3):164–74.

65. Wireko S, Asiedu SO, Kini P, Aglomasa BC, Amewu EKA, Asiedu E, et al. Prevalence of Methicillin-Resistant Staphylococcus Species Among Filarial Lymphedema Patients in Ahanta West District of Ghana. Front Trop Dis. 2021;2:786378.

66. Wolters M, Frickmann H, Christner M, Both A, Rohde H, Oppong K, et al. Molecular Characterization of Staphylococcus aureus Isolated from Chronic Infected Wound in Rural Ghana. Microorganisms. 2020;8 (12):2052.

67. Egyir B, Bentum J, Attram N, Fox A, Obeng-Nkrumah N, Appiah-Korang L, et al. Whole Genome Sequencing and Antimicrobial Resistance of Staphylococcus aureus from Surgical Site Infections in Ghana. Pathogens. 2021;10 (2):196.

68. Onuzo CN, Sefogah PE, Nuamah MA, Ntumy M, Osei MM, Nkyekyer K. Surgical site infections following caesarean sections in the largest teaching hospital in Ghana. Infect Prev Pract. 2022;4 (2):100203.

69. Sanders T, Bentum J, Fox A, Egyir B, Watters C. Characterization of MRSA and ESBL pathogens from patients with surgical-site infections in Accra, Ghana. Antimicrob Steward Healthc Epidemiol. 2022;2 (1):86.

70. Maïga A, Dicko OA, Tchougoune LM, Fofana DB, Coulibaly DM, Maïga II. High prevalence of methicillin-resistant Staphylococcus aureus strains in the Point G teaching hospital in Bamako, Mali. Mali Med. 2017;32 (3):1–8.

71. Dicko OA, Maïga A, Diarra B, Maïga II. Prevalence and antimicrobial susceptibility of the borderline oxacillin-resistant Staphylococcus aureus (BORSA) strains in Bamako, Mali. African J Bacteriol Res. 2023;15 (1):23–30.

72. Kalambry AC, Potindji TMF, Guindo I, Kassogue A, Kambire D, Dramé BSI, et al. Resistance phenotypes and molecular characteristics of Staphylococcus aureus associated with pleuritis in patients at “Hôpital du Mali” teaching hospital. Res Sq. 2024;:rs.3.rs-3579825.

73. Adetayo TO, Deji-Agboola AM, Popoola MY, Atoyebi TJ, Egberongbe KJ. Prevalence of methicillin resistant Staphylococcus aureus from clinical specimens in Ibadan, Nigeria. Int J Eng Sci. 2014;3 (9):1–11.

74. Alli O, Ogbolu D, Shittu A, Okorie A, Akinola J, Daniel J. Association of virulence genes with mecA gene in Staphylococcus aureus isolates from Tertiary Hospitals in Nigeria. Indian J Pathol Microbiol. 2015;58 (4):464–71.

75. Yusuf EO, Airauhi LU. Prevalence and pattern of methicillin resistant Staphylococcus aureus in a tertiary healthcare facility in Nigeria. Med J Zambia. 2015;42 (1):7–11.

76. Nsofor CA, Nwokenkwo VN, Ohale CU. Prevalence and antibiotic susceptibility pattern of Staphylococcus aureus isolated from various clinical specimens in south-East Nigeria. MOJ Cell Sci Rep. 2016;3 (2):1–5.

77. Osinupebi OA, Osiyemi JA, Deji-Agboola AM, Akinduti PA, Ejilude O, Makanjuola SO, et al. Prevalence of Methicillin-resistant Staphylococcus aureus in Abeokuta, Nigeria. South Asian J Res Microbiol. 2018;1 (1):1–8.

78. Chukwueze CM, Udeani TK, Obeagu EI, Ikpenwa JN, Nneka A. Prevalence of Methicillin Resistant Staphylococcus aureus Infections among Hospitalized Wound Patients from Selected Tertiary Hospitals within Enugu Metropolis. J Adv Med Pharm Sci. 2022;24 (3):18–27.

79. Udeani TK, Onyebuchi CJ, Ikpenwa MC, Ezenwaka UR. Prevalence and antibiotic susceptibility pattern of methicillin resistant Staphylococcus aureus in burns and pressure ulcer patients. African J Clin Exp Microbiol. 2016;17 (2):130–9.

80. Abdullahi N, Iregbu KC. Methicillin-Resistant Staphylococcus aureus in a Central Nigeria Tertiary Hospital. Ann Trop Pathol. 2018;9 (1):6–10.

81. AbdulAziz ZA, Onaolapo JA, Ibrahim Y, Olayinka B, Abdulaziz MM. View of Prevalence and Antimicrobial Resistance Profile of Methicillin Resistant Staphylococcus aureus isolates from Wound Infections in Zaria, Nigeria. J Curr Biomed Res. 2022;2 (5):475–89.

82. Olowo-Okere A, Atata RF, Abass A, Shuaibu AS, Yahya UH, Tanko N. Incidence and Antibiotic Susceptibility Profile of Staphylococcus aureus Isolates from Wound of Patients at Specialist Hospital, Sokoto, Nigeria. J Med Bacteriol. 2017;6 (3-4):44–50.

83. Mofolorunsho KC, Emmanuel MT, Omatola CA, Aminu RF, Ocheni HO. Prevalence and Antibiotic Resistance Profiles of Methicillin-Resistant Staphylococcus aureus Isolated from Clinical Specimens in Anyigba, Nigeria. UMYU J Microbiol Res. 2022;7 (1):38–46.

84. Beshiru A, Uwhuba KE. Molecular Identification and Antibiogram of Methicillin-Resistant Staphylococcus aureus from Wound of both In- And Out-patients at University of Benin Teaching Hospital (UBTH), Benin City, Nigeria. FUDMA J Sci. 2023;7 (3):323–31.

85. Medugu N, Nwajiobi-Princewill PI, Shettima SA, Mohammed MM, Mohammed Y, Wariso K, et al. A mini- national surveillance study of resistance profiles of Staphylococcus Aureus isolated from clinical specimens across hospitals in Nigeria. Niger J Clin Pract. 2021;24 (2):225–32.

86. Ibadin EE, Enabulele IO, Muinah F. Prevalence of mecA gene among staphylococci from clinical samples of a tertiary hospital in Benin City, Nigeria. Afr Health Sci. 2017;17 (4):1000–10.

87. Ganau AM, Manga SB. Methicillin Resistant Staphylococcus aureus (MRSA) in Hospitals of Sokoto Metropolis - A Multicentre Surveillance and Review of Literature. J Med Lab Sci. 2019;29 (2):21–36.

88. Olowe OA, Kukoyi OO, Taiwo SS, Ojurongbe O, Opaleye OO, Bolaji OS, et al. Phenotypic and molecular characteristics of methicillin-resistant Staphylococcus aureus isolates from Ekiti State, Nigeria. Infect Drug Resist. 2013;6:87–92.

89. Idris AM, Kumurya AS, Mohammed Y, Mustapha HM. Phenotypic determination of methicillin-resistant Staphylococcus aureus in Aminu Kano Teaching Hospital, Kano, Nigeria. Niger J Exp Clin Biosci. 2019;7 (1):1–6.

90. Oche DA, Oyegoke PI, Akpudo MO, Olayinka BO. Antibiotics Resistance Pattern of Staphylococcus aureus Isolated From In-patients of an Orthopaedic Hospital in North-Western Nigeria. Epidemiol Heal Syst J. 2021;8 (4):160–6.

91. Umar AI, Manga SB, Baki AS, Uba A. Molecular characterization and epidemiology of methicillin-resistant Staphylococcus aureus isolated from clinical samples in Sokoto, Nigeria. Adesh Univ J Med Sci Res. 2023;5 (1):17–24.

92. Okoye EL, Omeje MJ, Ugwuoji ET. Detection and prevalence of methicillin and vancomycin resistant staphylococcus aureus among clinical isolates in ESUTH, Enugu State. J Curr Biomed Res. 2022;2 (2):170–86.

93. Akanbi BO, Mbe JU. Occurrence of Methicillin and Vancomycin Resistant Staphylococcus aureus in University of Abuja Teaching Hospital, Abuja, Nigeria. African J Clin Exp Microbiol. 2013;14 (1):10–3.

94. Bawonda EO, Moses AE, Etang UE. Occurrence of high level methicillin resistance Staphylococcus aureus in patients from health facilities in Akwa Ibom State, Nigeria. Ibom Med J. 2024;17 (1):56–61.

95. Oba AN, Nuhu A. Prevalence and Antibiotics resistance profile of Staphylococcus aureus isolated from post-operative Wound in secondary health facilities of Ilorin metropolis, Kwara State, Nigeria. UMYU J Microbiol Res. 2023;8 (1):101–8.

96. Ejikeugwu C, Okike CE, Edeh C, Nwezeagu F, Ugwu M. Characterization of methicillin resistant Staphylococcus aureus (MRSA) isolates using oxacillin-cefoxitin disk diffusion test (OCDDT). Int Res J Public Heal. 2018;2:22.

97. Udobi CE, Obajuluwa AF, Onaolapo JA. Prevalence and Antibiotic Resistance Pattern of Methicillin-Resistant Staphylococcus aureus from an Orthopaedic Hospital in Nigeria. Biomed Res Int. 2013;2013 (1):860467.

98. Angel OD, Kadarko PS, Muazu JS, Bassey BE, Helma AR, Haruna NI, et al. Antimicrobial resistance profile and molecular detection of MecA gene in methicillin resistant Staphylococcus aureus from patients in selected general hospitals in Abuja municipal, Nigeria. GSC Biol Pharm Sci. 2019;7 (3):093–106.

99. Bunza NM, Isah AA, Hafsat MD, Asiya UI. Antibiotic Susceptibility Pattern of Staphylococcus aureus Isolated from Clinical Samples in Specialist Hospital, Sokoto. South Asian J Res Microbiol. 2019;3 (3):1–6.

100. Yahaya H, Ahmad AS, Ibrahim A, Abdullahi SA. Phenotypic Detection of Macrolide, Lincosamide and Streptogramin B Resistance Among Staphylococcus Aureus Clinical Isolates in A Northern Nigeria Tertiary Hospital. AlQalam J Med Appl Sci. 2022;5 (1):193–8.

101. Obasuyi O, Akerele J. High incidence of multidrug-resistant strains of methicill inresistant Staphylococcus aureus isolated from clinical samples in Benin-City, Nigeria. African J Clin Exp Microbiol. 2015;16 (3):124–7.

102. Schaumburg F, Vas Nunes J, Mönnink G, Falama A, Bangura J, Mathéron H, et al. Chronic Wound in Sierra Leone: pathogen spectrum and antimicrobial susceptibility. Infection. 2022;50 (4):907–14.

103. Dossim S, Bawe LD, Dossouvi KM, Maba D, Lawani A-A, Godonou AM, et al. Antibiotic Resistance Profiles of Bacteria Isolated from Patients Hospitalized at the Sylvanus Olympio University Teaching Hospital in Lomé, Togo. Microbiol Res J Int. 2024;34 (4):13–23.

104. Darboe S, Dobreniecki S, Jarju S, Jallow M, Mohammed NI, Wathuo M, et al. Prevalence of Panton-Valentine Leukocidin (PVL) and Antimicrobial Resistance in Community-Acquired Clinical Staphylococcus aureus in an Urban Gambian Hospital: A 11-year period retrospective pilot study. Front Cell Infect Microbiol. 2019;9:170.

105. Gouleu CS, Daouda MA, Oye Bingono SO, McCall MBB, Alabi AS, Adegnika AA, et al. Temporal trends of skin and soft tissue infections caused by methicillin-resistant Staphylococcus aureus in Gabon. Antimicrob Resist Infect Control. 2024;13 (1):1–6.

106. Alabi AS, Frielinghaus L, Kaba H, Kösters K, Huson MAM, Kahl BC, et al. Retrospective analysis of antimicrobial resistance and bacterial spectrum of infection in Gabon, Central Africa. BMC Infect Dis. 2013;13 (1):1–6.

107. Schaumburg F, Ngoa UA, Kösters K, Köck R, Adegnika AA, Kremsner PG, et al. Virulence factors and genotypes of Staphylococcus aureus from infection and carriage in Gabon. Clin Microbiol Infect. 2011;17 (10):1507–13.

108. Dikoumba A-C, Onanga R, Nguema PPM, Mangouka LG, Iroungou BA, Kassa FK, et al. Phenotipic Prevalence of Antibiotic Resistance in Gabon. Open J Med Microbiol. 2021;11 (2):100–18.

109. Okuda K V., Toepfner N, Alabi AS, Arnold B, Bélard S, Falke U, et al. Molecular epidemiology of Staphylococcus aureus from Lambaréné, Gabon. Eur J Clin Microbiol Infect Dis. 2016;35 (12):1963–73.

110. Vandendriessche S, De Boeck H, Deplano A, Phoba MF, Lunguya O, Falay D, et al. Characterisation of Staphylococcus aureus isolates from Bloodstream infections, Democratic Republic of the Congo. Eur J Clin Microbiol Infect Dis. 2017;36 (7):1163–71.

111. Iyamba JML, Wambale JM, Lukukula CM, Takaisi-Kikuni N za B. High prevalence of methicillin resistant staphylococci strains isolated from surgical site infections in Kinshasa. Pan Afr Med J. 2014;18:322.

112. Mohamadou M, Essama SR, Essome MCN, Akwah L, Nadeem N, Kamga HG, et al. High prevalence of Panton-Valentine leukocidin positive, multidrug resistant, Methicillin-resistant Staphylococcus aureus strains circulating among clinical setups in Adamawa and Far North regions of Cameroon. PLoS One. 2022;17 (7):e0265118.

113. Bissong MEA, Wirgham T, Enekegbe MA, Niba PTN, Foka FET. Prevalence and Antibiotic Susceptibility Patterns of Methicillin Resistant Staphylococcus Aureus in Patients Attending the Laquintinie Hospital Douala, Cameroon. Eur J Clin Biomed Sci. 2017;2 (6):92–6.

114. Foloum AK, Founou LL, Karang S, Maled Y, Tsayem C, Kuete M, et al. Methicillin Resistant Staphylococci Isolated in Clinical Samples: a 3-year Retrospective Study Analysis. Futur Sci OA. 2021;7 (4).

115. Manhafo VN, Tadongfack TD, Nitcheu ILS, Nkouayep VR, Selabi ACN, Kamtchueng MO, et al. Prevalence and Antimicrobial Resistance Pattern of Community Acquired Staphylococcus aureus in Patients Received at the Traumatology Unit of a Secondary Referral Health Setting in the Western Cameroon. Int J Pathog Res. 2021;8 (1):28–36.

116. Massongo M, Ngando L, Pefura Yone EW, Nzouankeu A, Mbanzouen W, Fonkoua MC, et al. Trends of Antibacterial Resistance at the National Reference Laboratory in Cameroon: Comparison of the Situation between 2010 and 2017. Biomed Res Int. 2021;2021 (1):9957112.

117. Kengne MF, Mbaveng AT, Kuete V. Antibiotic Resistance Profile of Staphylococcus aureus in Cancer Patients at Laquintinie Hospital in Douala, Littoral Region, Cameroon. Biomed Res Int. 2024;2024 (1):5859068.

118. Fonkoue L, Tissingh EK, Ngouateu MT, Muluem KO, Ngongang O, Mbouyap P, et al. The Microbiological Profile and Antibiotic Susceptibility of Fracture Related Infections in a Low Resource Setting Differ from High Resource Settings: A Cohort Study from Cameroon. Antibiotics. 2024;13 (3):236.

119. Ndedy MM, Nyasa RB, Esemu SN, Kfusi JA, Keneh NK, Masalla TN, et al. A cross-sectional study on the prevalence and drug susceptibility pattern of methicillin-resistant Staphylococcus aureus isolated from patients in the Buea Health District, Cameroon. Pan Afr Med J. 2023;45 (1).

120. Kengne M, Fotsing O, Ndomgue T, Nwobegahay JM. Antibiotic susceptibility patterns of Staphylococcus aureus strains isolated at the Yaounde Central Hospital, Cameroon: a retro prospective study. Pan Afr Med J. 2019;32 (1).

121. Gaké B, Bamia A, Mohamadou M, Tapindji NM, Mbakop CD, Essome MCN, et al. Methicillin-Resistant Staphylococcus aureus (MRSA): A major concern in the Northern Cameroon. GSC Biol Pharm Sci. 2022;20 (2):119–26.

122. Abdoul-Latif FM, Aboubaker IH, Abdoul-Latif HM, Ali AM, Ainane T. Staphylococcus aureus infections in patients from 2019–2021 at Peltier hospital of Djibouti. J Anal Sci Appl Biotechnol. 2022;4 (1):50–4.

123. Garoy EY, Gebreab YB, Achila OO, Tekeste DG, Kesete R, Ghirmay R, et al. Methicillin-Resistant Staphylococcus aureus (MRSA): Prevalence and Antimicrobial Sensitivity Pattern among Patients—A Multicenter Study in Asmara, Eritrea. Can J Infect Dis Med Microbiol. 2019;2019:8321834.

124. Garoy EY, Gebreab YB, Achila OO, Tecklebrhan N, Tsegai HM, Hailu AZ, et al. Magnitude of Multidrug Resistance among Bacterial Isolates from Surgical Site Infections in Two National Referral Hospitals in Asmara, Eritrea. Int J Microbiol. 2021;2021 (1):6690222.

125. Kahsay A, Mihret A, Abebe T, Andualem T. Isolation and antimicrobial susceptibility pattern of Staphylococcus aureus in patients with surgical site infection at Debre Markos Referral Hospital, Amhara Region, Ethiopia. Arch Public Heal. 2014;72 (1):1–7.

126. Godebo G, Kibru G, Tassew H. Multidrug-resistant bacterial isolates in infected Wound at Jimma University Specialized Hospital, Ethiopia. Ann Clin Microbiol Antimicrob. 2013;12 (1):1–7.

127. Mitiku A, Aklilu A, Biresaw G, Gize A. Prevalence and associated factors of methicillin resistance staphylococcus aureus (MRSA) among urinary tract infection suspected patients attending at arba minch general hospital, southern ethiopia. Infect Drug Resist. 2021;14:2133–42.

128. Tsige Y, Tadesse S, G/Eyesus T, Tefera MM, Amsalu A, Menberu MA, et al. Prevalence of Methicillin-Resistant Staphylococcus aureus and Associated Risk Factors among Patients with Wound Infection at Referral Hospital, Northeast Ethiopia. J Pathog. 2020;2020 (1):3168325.

129. Dilnessa T, Bitew A. Prevalence and antimicrobial susceptibility pattern of methicillin resistant Staphylococcus aureus isolated from clinical samples at Yekatit 12 Hospital Medical College, Addis Ababa, Ethiopia. BMC Infect Dis. 2016;16 (1):1–9.

130. Kejela T, Dekosa F. High prevalence of MRSA and VRSA among Inpatients of Mettu Karl Referral Hospital, Southwest Ethiopia. Trop Med Int Heal. 2022;27 (8):735–41.

131. Worku S, Abebe T, Seyoum B, Alemu A, Shimelash Y, Yimer M, et al. Molecular Epidemiology of Methicillin-Resistant Staphylococcus aureus among Patients Diagnosed with Surgical Site Infection at Four Hospitals in Ethiopia. Antibiotics. 2023;12 (12):1681.

132. Tefera S, Awoke T, Mekonnen D. Methicillin and vancomycin resistant staphylococcus aureus and associated factors from surgical ward Inpatients at debre markos referral hospital, northwest ethiopia. Infect Drug Resist. 2021;14:3053–62.

133. Tadesse S, Alemayehu H, Tenna A, Tadesse G, Tessema TS, Shibeshi W, et al. Antimicrobial resistance profile of Staphylococcus aureus isolated from patients with infection at Tikur Anbessa Specialized Hospital, Addis Ababa, Ethiopia. BMC Pharmacol Toxicol. 2018;19 (1):1–8.

134. Moges F, Tamiru T, Amare A, Mengistu G, Eshetie S, Dagnew M, et al. Prevalence of Methicillin-ResistantStaphylococcus aureus and Multidrug-Resistant Strains from Patients Attending the Referral Hospitals of Amhara Regional State, Ethiopia. Int J Microbiol. 2023;2023 (1):3848073.

135. Asefa M, Tigabu E, Fentaw S, Abubekure R, Mehiret A, Asamene N, et al. Prevalence of methicillin resistant Staphylococcus aureus in patient specimens referred to National Reference Laboratory, Ethiopian Public Health Institute. Ethiop J Public Heal Nutr. 2019;3 (1):34–8.

136. Abebe M, Alemkere G, Ayele G. Methicillin and vancomycin-resistant Staphylococcus aureus and associated risk factors among patients with Wound infection in East Wallaga Zone, Western Ethiopia. Infect Prev Pract. 2024;6 (4):100409.

137. Mama M, Aklilu A, Misgna K, Tadesse M, Alemayehu E. Methicillin- and Inducible Clindamycin-Resistant Staphylococcus aureus among Patients with Wound Infection Attending Arba Minch Hospital, South Ethiopia. Int J Microbiol. 2019;2019 (1):2965490.

138. Wangai FK, Masika MM, Maritim MC, Seaton RA. Methicillin-resistant Staphylococcus aureus (MRSA) in East Africa: Red alert or red herring?. BMC Infect Dis. 2019;19 (1):1–10.

139. Akoru C, Kuremu RT, Ndege SK, Obala A, Smith JW, Bartlett M, et al. Prevalence and Anti-Microbial Susceptibility of Methicillin Resistant Staphylococcus aureus at Moi Teaching and Referral Hospital Eldoret. Open J Med Microbiol. 2016;6 (1):9–16.

140. Gitau W, Masika M, Musyoki M, Museve B, Mutwiri T. Antimicrobial susceptibility pattern of Staphylococcus aureus isolates from clinical specimens at Kenyatta National Hospital. BMC Res Notes. 2018;11:1–5.

141. Iliya S, Mwangi J, Maathai R, Muriuki M. Phenotypic analysis and antibiotic susceptibility of methicillin-resistant Staphylococcus aureus in Kiambu County, Kenya. J Infect Dev Ctries. 2020;14 (6):597–605.

142. Okello C, Ombajo L, Omonge E, Otieno F, Otieno D, Mwachari C. Antimicrobial susceptibility patterns of Staphylococcus aureus in a tertiary referral hospital in Nairobi, Kenya. Int J Med Med Sci. 2021;13 (2):22–7.

143. Omuse G, Kabera B, Revathi G. Low prevalence of methicillin resistant Staphylococcus aureus as determined by an automated identification system in two private hospitals in Nairobi, Kenya: a cross sectional study. BMC Infect Dis. 2014;14 (1):1–6.

144. Maina EK, Kiiyukia C, Wamae CN, Waiyaki PG, Kariuki S. Characterization of methicillin-resistant Staphylococcus aureus from skin and soft tissue infections in patients in Nairobi, Kenya. Int J Infect Dis. 2013;17 (2):115–9.

145. Musicha P, Cornick JE, Bar-Zeev N, French N, Masesa C, Denis B, et al. Trends in antimicrobial resistance in Bloodstream infection isolates at a large urban hospital in Malawi (1998–2016): a surveillance study. Lancet Infect Dis. 2017;17 (10):1042–52.

146. Choonara FE, Haldorsen BC, Ndhlovu I, Saulosi O, Maida T, Lampiao F, et al. Antimicrobial susceptibility profiles of clinically important bacterial pathogens at the Kamuzu Central Hospital in Lilongwe, Malawi. Malawi Med J. 2022;34 (1):9–16.

147. Nuckchady DC, Boolaky SH. The Prevalence of Multi-Drug Resistant Organisms and Their Outcomes in an ICU in Mauritius: An Observational Study. Asian J Med Heal. 2020;18 (11):71–8.

148. Vubil D, Garrine M, Ruffing U, Acácio S, Sigaúque B, Alonso PL, et al. Molecular characterization of community acquired Staphylococcus aureus bacteremia in young children in southern Mozambique, 2001-2009. Front Microbiol. 2017;8:248470.

149. Kenga DB, Gebretsadik T, Simbine S, Maússe FE, Charles P, Zaqueu E, et al. Community-acquired bacteremia among HIV-infected and HIV-exposed uninfected children hospitalized with fever in Mozambique. Int J Infect Dis. 2021;109:99–107.

150. Van der Meeren BT, Millard PS, Scacchetti M, Hermans MH, Hilbink M, Concelho TB, et al. Emergence of methicillin resistance and Panton-Valentine leukocidin positivity in hospital- and community-acquired Staphylococcus aureus infections in Beira, Mozambique. Trop Med Int Heal. 2014;19 (2):169–76.

151. Garrine M, Costa SS, Messa A, Massora S, Vubil D, Ácacio S, et al. Antimicrobial resistance and clonality of Staphylococcus aureus causing bacteraemia in children admitted to the Manhiça District Hospital, Mozambique, over two decades. Front Microbiol. 2023;14:1208131.

152. Ishimwe E, Rogo T. Antibiotic Resistance in Children with Bacteremia Admitted in the Largest Tertiary Hospital in Rwanda. Rwanda Med J. 2018;75 (2):5–8.

153. Masaisa F, Kayigi E, Seni J, Bwanga F, Muvunyi CM. Antibiotic Resistance Patterns and Molecular Characterization of Methicillin-Resistant Staphylococcus aureus in Clinical Settings in Rwanda. Am J Trop Med Hyg. 2018;99 (5):1239–45.

154. Ntirenganya C, Manzi O, Muvunyi CM, Ogbuagu O. High prevalence of antimicrobial resistance among common bacterial isolates in a tertiary healthcare facility in Rwanda. Am J Trop Med Hyg. 2015;92 (4):865–70.

155. Sutherland T, Mpirimbanyi C, Nziyomaze E, Niyomugabo JP, Niyonsenga Z, Muvunyi CM, et al. Widespread antimicrobial resistance among bacterial infections in a Rwandan referral hospital. PLoS One. 2019;14 (8):e0221121.

156. Noel G, Benigne B, Vincent M. Prevalence of Staphylococcus aureus among Clinical Isolates and their Responses to Selected Antibiotics at Centre Hospitalier Universitaire de Kigali (CHUK). J Microbiol Exp. 2017;5 (4).

157. Kumburu HH, Sonda T, Leekitcharoenphon P, Van Zwetselaar M, Lukjancenko O, Alifrangis M, et al. Hospital Epidemiology of Methicillin-Resistant Staphylococcus aureus in a Tertiary Care Hospital in Moshi, Tanzania, as Determined by Whole Genome Sequencing. Biomed Res Int. 2018;2018 (1):2087693.

158. Manyahi J, Matee MI, Majigo M, Moyo S, Mshana SE, Lyamuya EF. Predominance of multi-drug resistant bacterial pathogens causing surgical site infections in Muhimbili National Hospital, Tanzania. BMC Res Notes. 2014;7 (1):1–7.

159. Onken A, Said AK, Jørstad M, Jenum PA, Blomberg B. Prevalence and Antimicrobial Resistance of Microbes Causing Bloodstream Infections in Unguja, Zanzibar. PLoS One. 2015;10 (12):e0145632.

160. Ernest R, Lema N, Yassin S, Joachim A, Majigo M. Bacterial aetiology, antimicrobial susceptibility patterns, and factors associated with urinary tract infection among under-five children at primary health facility, North-Western Tanzania. PLoS One. 2024;19 (5):e0303369.

161. Moremi N, Claus H, Mshana SE. Antimicrobial resistance pattern: A report of microbiological cultures at a tertiary hospital in Tanzania. BMC Infect Dis. 2016;16 (1):1–7.

162. Kamori D, Renatus D, Mwandigha AM, Emmanuel E, Masoud SS, Shedura V, et al. Prevalence and patterns of multidrug-resistant bacteria isolated from Sputum samples of patients with bacterial pneumonia at a tertiary hospital in Tanzania. Bull Natl Res Cent. 2024;48 (1):1–8.

163. Mwailunga HA, Katemi ES, Niccodem EM, Matee MI. Prevalence of methicillin and clindamycin resistant Staphylococcus species at a tertiary hospital in Tanzania: Implications for antibiotic stewardship and infection management. Ger J Microbiol. 2023;3 (3):1–6.

164. Mnyambwa NP, Mahende C, Wilfred A, Sandi E, Mgina N, Lubinza C, et al. Antibiotic susceptibility patterns of bacterial isolates from routine clinical specimens from referral hospitals in tanzania: A prospective hospital-based observational study. Infect Drug Resist. 2021;14:869–78.

165. Geofrey MA, Sauli E, Kanje LE, Beti M, Shayo MJ, Kuchaka D, et al. Genomic characterization of methicillin-resistant Staphylococcus aureus isolated from patients attending regional referral hospitals in Tanzania. BMC Med Genomics. 2024;17:1–12.

166. Moremi N, Mushi MF, Fidelis M, Chalya P, Mirambo M, Mshana SE. Predominance of multi-resistant gram-negative bacteria colonizing chronic lower limb ulcers (CLLUs) at Bugando Medical Center. BMC Res Notes. 2014;7 (1):1–4.

167. Mushi MF, Mwalutende AE, Gilyoma JM, Chalya PL, Seni J, Mirambo MM, et al. Predictors of disease complications and treatment outcome among patients with chronic suppurative otitis media attending a tertiary hospital, Mwanza Tanzania. BMC Ear Nose Throat Disord. 2016;16 (1):1–8.

168. Johnson B, Stephen BM, Joseph N, Asiphas O, Musa K, Taseera K. Prevalence and bacteriology of culture-positive urinary tract infection among pregnant women with suspected urinary tract infection at Mbarara regional referral hospital, South-Western Uganda. BMC Pregnancy Childbirth. 2021;21 (1):1–9.

169. Pius T, Irege R, Makeri D, Tamale A. Methicillin-Resistant Staphylococcus aureus among Patients with Skin and Soft Tissue Infections: A Cross-Sectional Study at a Tertiary Hospital in Bushenyi, Western, Uganda. Open Access Libr J. 2023;10 (5):1–12.

170. George M, Iramiot JS, Muhindo R, Olupot-Olupot P, Nanteza A. Bacterial Aetiology and Antibiotic Susceptibility Profile of Post-Operative Sepsis among Surgical Patients in a Tertiary Hospital in Rural Eastern Uganda. Microbiol Res J Int. 2018;24 (2):41690.

171. Wekesa YN, Namusoke F, Sekikubo M, Mango DW, Bwanga F. Ceftriaxone- and ceftazidime-resistant Klebsiella species, Escherichia coli, and methicillin-resistant Staphylococcus aureus dominate caesarean surgical site infections at Mulago Hospital, Kampala, Uganda. SAGE Open Med. 2020;8:1–12.

172. Andrew B, Aidah K, Bazira JB. Prevalence of Methicillin Resistant Staphylococcus aureus among Isolates from Wound in Surgical Wards at Kabale Regional Referral Hospital, South Western Uganda. Microbiol Res J Int. 2016;17 (5):1–5.

173. Seni J, Bwanga F, Najjuka CF, Makobore P, Okee M, Mshana SE, et al. Molecular characterization of Staphylococcus aureus from patients with surgical site infections at Mulago Hospital in Kampala, Uganda. PLoS One. 2013;8 (6):e66153.

174. Stanley IJ, Bwanga F, Itabangi H, Nakaye M, Bashir M, Bazira J, et al. Prevalence and Antibiotic Susceptibility Patterns of Clinical Isolates of Methicillin-Resistant Staphylococcus aureus in a Tertiary Care Hospital in Western Uganda. Microbiol Res J Int. 2014;4 (10):1168–77.

175. Mwansa TN, Kamvuma K, Mulemena JA, Phiri CN, Chanda W. Antibiotic susceptibility patterns of pathogens isolated from laboratory specimens at Livingstone Central Hospital in Zambia. PLOS Glob Public Heal. 2022;2 (9):e0000623.

176. Shawa M, Paudel A, Chambaro H, Kamboyi H, Nakazwe R, Alutuli L, et al. Trends, patterns and relationship of antimicrobial use and resistance in bacterial isolates tested between 2015–2020 in a national referral hospital of Zambia. PLoS One. 2024;19 (4):e0302053.

177. Samutela TM. Molecular characterisation of methicillin-resistant staphylococcus aureus isolated at the Univeristy Teaching Hospital, Lusaka. Pan Afr Med J. 2015;26.

178. Roth BM, Laps A, Yamba K, Heil EL, Johnson JK, Stafford K, et al. Antibiogram Development in the Setting of a High Frequency of Multi-Drug Resistant Organisms at University Teaching Hospital, Lusaka, Zambia. Antibiotics. 2021;10 (7):782.

179. Simango C, Mulea L. Prevalence and antimicrobial susceptibility of methicillin resistant Staphylococcus aureus isolated from clinical specimens in Harare, Zimbabwe. Cent Afr J Med. 2019;65 (3):1–5.

180. Mauchaza K, Madzimbamuto FD, Waner S. Methicillin-resistant Staphylococcus aureus in Zimbabwe. Ghana Med J. 2016;50 (2):68–71.

181. Iileka AEK, Mukesi M, Engelbrecht F, Moyo SR. Antimicrobial Susceptibility Patterns of Staphylococcus aureus Strains Isolated at the Namibia Institute of Pathology from 2012 to 2014. Open J Med Microbiol. 2016;6 (3):116–24.

182. Naidoo R, Nuttall J, Whitelaw A, Eley B. Epidemiology of Staphylococcus aureus Bacteraemia at a Tertiary Children’s Hospital in Cape Town, South Africa. PLoS One. 2013;8 (10):e78396.

183. Oosthuysen WF, Orth H, Lombard CJ, Sinha B, Wasserman E. Population structure analyses of Staphylococcus aureus at Tygerberg Hospital, South Africa, reveals a diverse population, a high prevalence of Panton–Valentine leukocidin genes, and unique local methicillin-resistant S. aureus clones. Clin Microbiol Infect. 2014;20 (7):652–9.

184. Abdulgader SM, Lentswe T, Whitelaw A, Newton-Foot M. The prevalence and molecular mechanisms of mupirocin resistance in Staphylococcus aureus isolates from a Hospital in Cape Town, South Africa. Antimicrob Resist Infect Control. 2020;9 (1):1–7.

185. Perovic O, Singh-Moodley A, Govender NP, Kularatne R, Whitelaw A, Chibabhai V, et al. A small proportion of community-associated methicillin-resistant Staphylococcus aureus bacteraemia, compared to healthcare-associated cases, in two South African provinces. Eur J Clin Microbiol Infect Dis. 2017;36 (12):2519–32.

186. Shuping LL, Kuonza L, Musekiwa A, Iyaloo S, Perovic O. Hospital-associated methicillin-resistant Staphylococcus aureus: A cross-sectional analysis of risk factors in South African tertiary public hospitals. PLoS One. 2017;12 (11):e0188216.

187. Perovic O, Iyaloo S, Kularatne R, Lowman W, Bosman N, Wadula J, et al. Prevalence and Trends of Staphylococcus aureus Bacteraemia in Hospitalized Patients in South Africa, 2010 to 2012: Laboratory-Based Surveillance Mapping of Antimicrobial Resistance and Molecular Epidemiology. PLoS One. 2015;10 (12):e0145429.

188. Singh-Moodley A, Perovic O. Characterisation of Staphylococcus aureus Bloodstream isolates from Gauteng and Western cape provinces, South Africa, 2016 and 2017. Public Heal Surveill Bull. 2018;16 (2):99–106.

189. Fortuin-de Smidt MC, Singh-Moodley A, Badat R, Quan V, Kularatne R, Nana T, et al. Staphylococcus aureus bacteraemia in Gauteng academic hospitals, South Africa. Int J Infect Dis. 2015;30:41–8.

190. Strasheim W, Perovic O, Singh-Moodley A, Kwanda S, Ismail A, Lowe M. Ward-specific clustering of methicillin-resistant Staphylococcus aureus spa-type t037 and t045 in two hospitals in South Africa: 2013 to 2017. PLoS One. 2021;16 (6):e0253883.

191. Goolam Mahomed T, Kock MM, Masekela R, Hoosien E, Ehlers MM. Genetic relatedness of Staphylococcus aureus isolates obtained from cystic fibrosis patients at a tertiary academic hospital in Pretoria, South Africa. Sci Rep. 2018;8 (1):1–6.
